# D‐glucuronyl C5‐Epimerase Binds to EGFR to Suppress Kidney Fibrosis

**DOI:** 10.1002/advs.202416216

**Published:** 2025-08-11

**Authors:** Xiaoqi Jing, Jun Wu, Jingru Ning, Xiaoyu Ding, Zhenyun Du, Xiaojiang Wang, Lulin Huang, Ran Wang, Changlin Mei, Kan Ding

**Affiliations:** ^1^ Carbohydrate‐Based Drug Research Center CAS Key Laboratory of Receptor Research State Key Laboratory of Drug Research Shanghai Institute of Materia Medica Chinese Academy of Sciences Shanghai 201203 China; ^2^ Department of Nephrology Changzheng Hospital Second Military Medical University Shanghai 201203 China; ^3^ School of Chinese Materia Medica Nanjing University of Chinese Medicine Nanjing 210023 China; ^4^ Drug Discovery and Design Center State Key Laboratory of Drug Research Shanghai Institute of Materia Medica Chinese Academy of Sciences Shanghai 201203 China; ^5^ Department of Natural Medicine School of Pharmacy Fudan University Shanghai 201203 China; ^6^ Zhongshan Institute for Drug Discovery Shanghai Institute of Materia Medica Chinese Academy of Science Zhongshan 528400 China

**Keywords:** epidermal growth factor receptor, epithelial‐to‐mesenchymal transition, glucuronyl C5‐epimerase, kidney fibrosis

## Abstract

Renal tubular cells actively participate in fibrosis, leading to end‐stage renal failure. However, the key molecules involved in fibrogenesis remain unclear. Glucuronyl C5‐epimerase (*Hsepi*, gene name, *Glce*) is a key enzyme that catalyzes the biosynthesis of heparan sulfate (HS) chains attached to HS proteoglycans that are ubiquitously located on the cell membrane. Homozygous *Glce*‐/‐ mice may exhibit embryonic lethality and multi‐organ defects. However, the role of *Glce* in kidney fibrosis remains unclear. This study investigated the contribution of *Glce* to kidney development and its role in renal fibrosis pathogenesis. Here, it shows that *Glce* expression is significantly attenuated in the kidneys of patients with renal fibrosis and in animal models. Renal tubular‐specific *Glce* deletion in mice exacerbated kidney fibrosis, while AAV‐mediated *Glce* overexpression in unilateral ureteral obstruction‐treated mice ameliorated kidney fibrosis via the TGF‐β/Smad2/3 signaling pathway. Mechanistic studies indicate that *Glce* protein may bind to epidermal growth factor receptor (EGFR) to inactivate EGFR/ERK signaling and further impede TGF‐β/Smad signaling pathway and renal fibrosis in *Glce*‐/‐ and wild‐type mice. Notably, the anti‐fibrotic function is independent of *Glce* enzymatic activation. These findings reveal a novel function of *Glce*, which plays a key role in kidney fibrosis.

## Graphical abstract

Glucuronyl C5‐epimerase (*Hsepi, gene* name *Glce*) is a key enzyme catalyzing heparan sulfate (HS) chain biosynthesis in HS proteoglycans, which are ubiquitously expressed on cell membranes. In normal renal tubular cells, *Glce* binds to the intracellular domains of EGFR, maintaining stable and orderly EGFR phosphorylation. However, tubule‐specific *Glce* loss activates EGFR signaling and the TGF‐β‐Smad cascade, exacerbating tubulointerstitial injury and fibrosis. Notably, anti‐fibrotic function of *Glce* is independent of its enzymatic activity. The nephroprotective role of *Glce* underscores its critical function in renal fibrosis and inspires new therapeutic potential targets for chronic kidney disease (CKD) treatment.

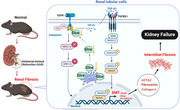

## 1 Introduction

Chronic kidney disease (CKD) affects one in ten people and is projected to be the fifth leading cause of years of life lost worldwide by 2040.^[^ ^1^ ^]^ Renal fibrosis, particularly tubulointerstitial fibrosis, is a common feature in most progressive CKDs and a major determinant of renal insufficiency without treatment.^[^ ^2^ ^]^ During the progression of CKD, extracellular matrix (ECM) accumulation accelerates disease advancement and is considered a reliable predictor of prognosis; however, the underlying mechanisms remain poorly understood.

Heparan sulfate (HS) is a linear glycan chain of HS proteoglycans (HSPGs) that is ubiquitously expressed on cell membranes and in the ECM of all tissues.^[^ ^3^ ^]^ HS chains are required for viral infections such as SARS‐Cov‐2,^[^ ^4^, ^5^ ^]^ and work as co‐receptors for fibroblast growth factors linked to the pathogenesis of various metabolic diseases, including obesity, non‐alcoholic steatohepatitis, primary biliary cirrhosis, and CKD.^[^ ^3^ ^]^ HS plays a pivotal role in the fibrosis linked to chronic allograft dysfunction through binding growth factors.^[^ ^6^ ^]^ The HS degradation enzyme, heparanase^[^ ^7^ ^]^ or modification enzyme glucosaminyl‐6‐O‐sulfotransferases functions in chronic renal fibrosis.^[^ ^8^ ^]^

Glucuronyl C5‐epimerase (*Hsepi*, gene name: *Glce*) is a modified enzyme involved in the biosynthesis of heparin and HS. It catalyzes the C5‐epimerization of the HS component, d‐glucuronic acid, into l‐iduronic acid, which provides internal flexibility to the polymer and forges protein‐binding sites to ensure polymer function.^[^ ^9^ ^]^ *Glce* is a highly conserved enzyme widely present between species and in various organs, including the brain, lungs, and kidneys.^[^ ^10^ ^]^ Previous studies indicated that targeted interruption of *Glce* in mice results in neonatal lethality accompanied by kidney agenesis, premature lung, and skeletal malformations, demonstrating that a single gene‐coded enzyme is essential for animal development.^[^ ^11^, ^12^ ^]^ Recently, we found that hepatic *Glce* deficiency led to impaired thermogenesis in adipose tissue and exacerbated high‐fat diet (HFD)‐induced obesity.^[^ ^13^ ^]^

Despite its implications in the growth and development of various organs in animals, the exact physiological function of *Glce* remains largely unknown. Combined with previous studies suggesting that all *Glce* ^‐/‐^ mice lack kidneys and show no overt abnormalities in other abdominal organs, while HS structural alteration by its biosynthesis or degradation enzymes has an impact on chronic kidney fibrosis,^[^ ^7^, ^8^ ^]^ we hypothesized that *Glce* might have a role in renal fibrosis. This study investigates the contribution of *Glce* to kidney development and its role in renal fibrosis pathogenesis. Using renal tubular epithelial cells and conditional knockout mice with clinical data from human renal tissues, we demonstrate that *Glce* deficiency enhances epithelial‐to‐mesenchymal transition (EMT) in renal tubular epithelial cells in vitro. Genetic ablation of epithelial *Glce* aggravated fibrosis through the EGFR/ERK pathway in unilateral ureteral obstruction (UUO)‐induced fibrosis models. Furthermore, we demonstrate that renal‐specific adeno‐associated viral (AAV)‐mediated *Glce* overexpression improves kidney fibrosis, independent of its catalytic isomerase activity. Our findings aim to reveal a new mechanism underlying the regulation of *Glce* activity during renal fibrosis and highlight the significance of preserving basal *Glce* levels in the kidney as a therapeutic strategy to attenuate the progression of kidney fibrosis.

## 2 Results

### 2.1 *Glce* Expression was Significantly Downregulated in Human and Mouse Fibrotic Kidneys

**Figure 1:**
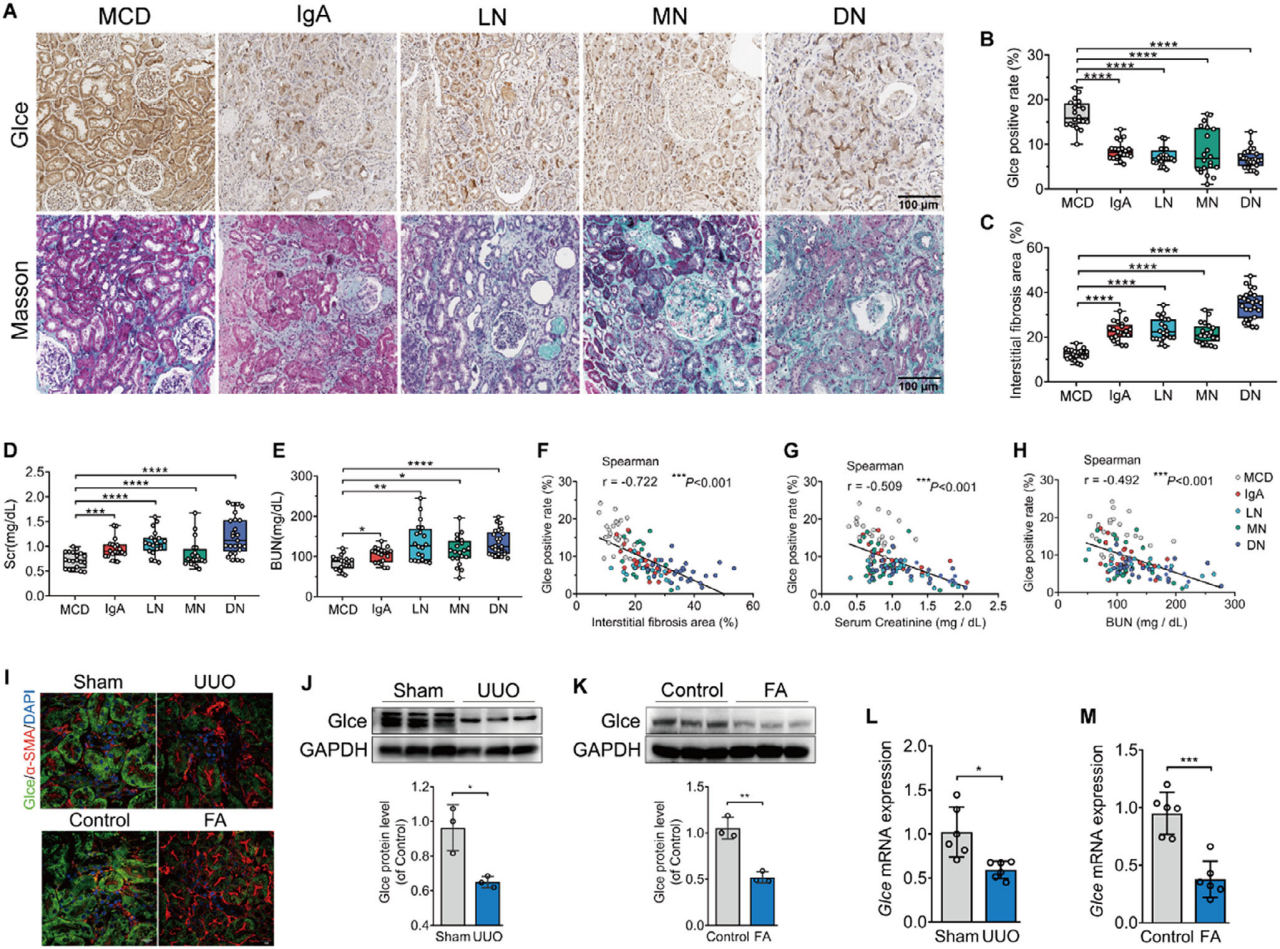
*Glce* expression was significantly downregulated in human and mouse fibrotic kidneys. A) Representative photomicrographs of *Glce* using immunohistochemical (IHC) and Masson staining in human renal tissues from patients with minimal change disease (MCD, *n*=20), IgA nephropathy (IgA, *n*=20), lupus nephritis (LN, *n*=20), membranous nephropathy (MN, n=20), and diabetic nephropathy (DN, *n*=25). Scale bars: 100 µm. B) Positivity rate of Glce protein with IHC staining and C) interstitial fibrosis area in renal biopsies from patients with different forms of chronic kidney disease (CKD). D) Serum creatinine (Scr) and E) blood urea nitrogen (BUN) in patients with different CKDs. F–H) Negative correlation between Glce protein positivity rate with IHC staining and interstitial fibrosis area (Spearman r = ‐ 0.722, *P *< 0.001), Scr (Spearman r = ‐ 0.509, *P *< 0.001), and BUN (Spearman r = ‐ 0.492, *P *< 0.001) in all patients. I) Immunofluorescent staining of Glce (green) and α‐SMA (red) in kidneys of unilateral ureteral obstruction (UUO)‐operated and folic acid (FA)‐treated mice. Scale bars: 20 µm. J) Immunoblots showing protein expression levels of Glce in whole‐kidney extracts from wild‐type (WT) mice 14 days after UUO (*n* = 6). K) Immunoblots showing the protein expression levels of Glce in whole‐kidney extracts from WT mice one month after a single intraperitoneal injection of FA (250 mg kg^−1^ body weight) (*n* = 6). L,M) *Glce* mRNA expression in whole‐kidney extracts from WT mice following UUO or FA treatment (*n* = 6). GAPDH was used as a loading control. Data are presented as mean ± standard error of the mean (SEM). ^*^ *P* < 0.05; ^**^ *P *< 0.01; ^***^ *P* < 0.001; ^****^ *P* < 0.0001 by unpaired, 2‐tailed Student's *t*‐test (J–M), one‐way ANOVA with Dunnett's *post hoc* tests (B, C, and E), Spearman's correlation test (F–H), and Kruskal‐Wallis test (D).

To investigate the potential involvement of *Glce* in the pathogenesis of kidney fibrosis, we examined *Glce* in renal biopsies from patients with IgA nephropathy (IgA), lupus nephritis (LN), membranous nephropathy (MN), and diabetic nephropathy (DN), comparing them with those with minimal change disease (MCD) using immunohistochemistry (IHC) staining (**Figure** **1**A). Notably, the Glce positivity rate was markedly reduced in renal biopsies from patients with different CKDs compared with MCD (Figure 1B). Additionally, patients with advanced CKD showed a more accentuated decline in Glce positivity rate, except in the LN group (Figure S1A–H, Supporting Information). We observed that patients with MCD had significantly less interstitial fibrosis than those with other types of CKD (Figure 1A,C). To assess the severity of kidney injury, we measured the serum creatinine (Scr) and blood urea nitrogen (BUN) levels as markers of renal function.^[^ ^14^ ^]^ The results showed that Scr and BUN levels in the MCD group were significantly lower than those in the other groups (Figure 1D,E). Furthermore, we found a negative correlation between Glce positivity rate and interstitial fibrosis area (Figure 1F) as well as Scr (Figure 1G) and BUN levels in all patients (Figure 1H). Considering the significant association of *Glce* decline with nearly all prevalent CKD cases, we analyzed mice subjected to UUO or folic acid (FA) treatment. In these mouse models, *Glce* mRNA and protein levels reduced over time (Figure 1J–M; Figure S2B,C, Supporting Information). Furthermore, immunofluorescence showed a significant reduction of Glce expression accompanied by an increase in α‐SMA levels^[^ ^15^ ^]^ in the kidneys of UUO and FA‐treated mice (Figure 1I).

### 2.2 *Glce* Deficiency Aggravated Renal Failure, and Renal‐Specific AAV‐Mediated *Glce* Overexpression Improved Kidney Fibrosis

**Figure 2:**
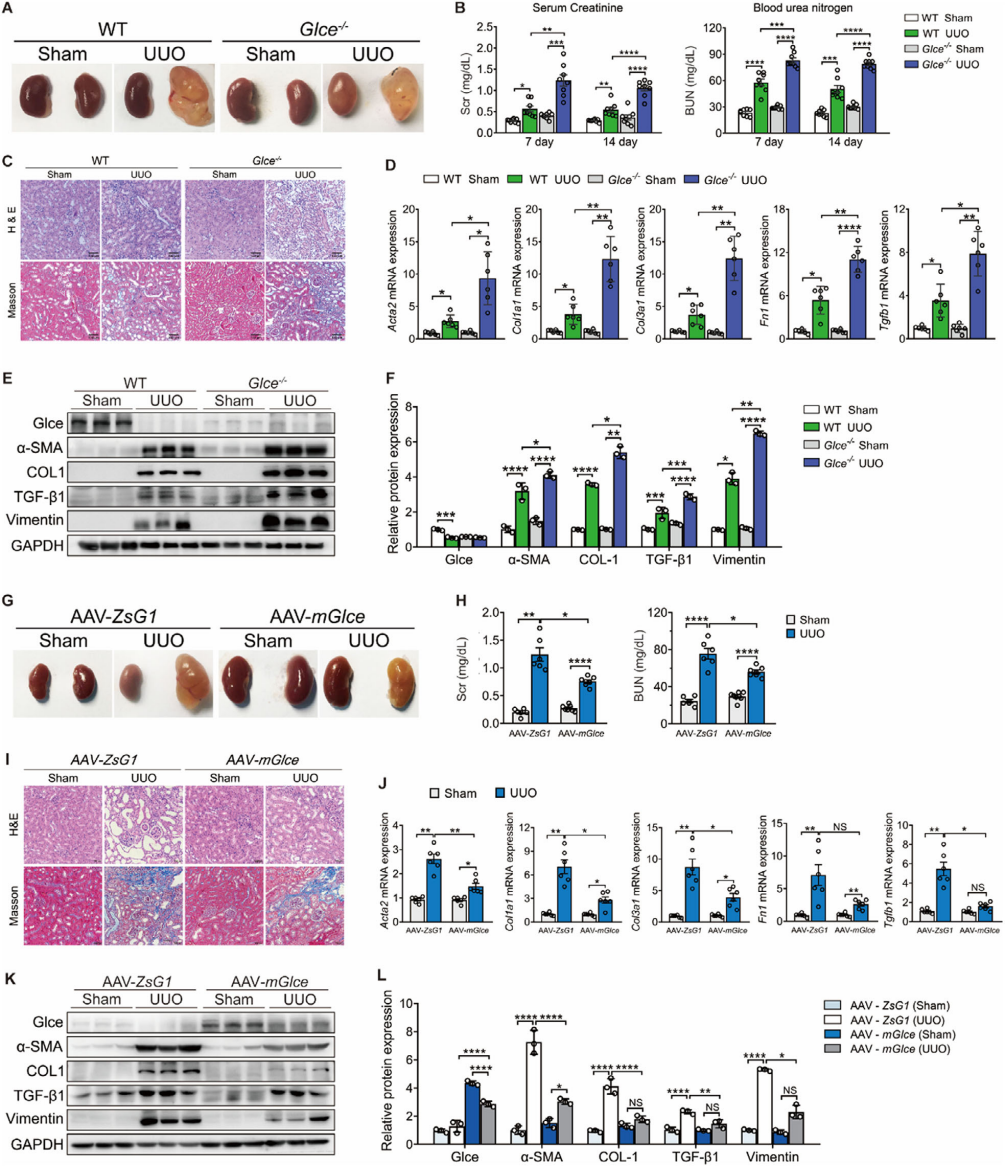
*Glce* deficiency aggravated renal failure, and renal‐specific AAV‐mediated *Glce* overexpression improved kidney fibrosis. A) Representative images of WT and *Glce* ^‐/‐^ mice kidneys after sham or UUO treatment taken at 12 weeks of age. B) Scr and BUN levels in WT and *Glce* ^‐/‐^ mice 7 and 14 days after sham or UUO treatment (*n* = 8). C) Representative hematoxylin and eosin (H&E) and Masson staining of kidney sections from WT and *Glce* ^‐/‐^ mice 14 days after sham or UUO treatment. Scale bar: 100 µm. D) mRNA and E,F) protein expression levels of fibrosis markers in WT and *Glce* ^‐/‐^ mice 14 days after sham or UUO treatment (*n* = 3–6). G) Morphological changes of kidneys in *Glce* ^‐/‐^ mice with the sham or UUO treatment after adeno‐associated viral (AAV) encoding vector plasmid (AAV‐ZsG1) or full‐length mouse *Glce* (AAV‐m*Glce*) injection. H) Scr and BUN levels of AAV‐ZsG1 and AAV‐m*Glce* mice after sham or UUO treatment (*n* = 6–7) I) Representative H&E staining, Masson's trichrome staining of *Glce* ^‐/‐^ mice kidneys with sham or UUO treatment after the AAV injection. Scale bar: 100 µm. J) mRNA and K,L) protein levels of fibrosis markers in AAV‐ZsG1 and AAV‐m*Glce* mice after sham or UUO treatment (n = 6). GAPDH was used as a loading control. Data are presented as mean ± SEM. ^*^ *P* < 0.05; ^**^ *P *< 0.01; ^***^ *P* < 0.001; ^****^ *P* < 0.0001 by one‐way ANOVA with Tukey or Dunnett's *post hoc* tests (B,D,F,H,J, and L).

To determine *Glce* expression and distribution in the kidney, kidney samples from patients and mice were stained. The results showed that *Glce* was predominantly present in tubular cells, with fewer levels in interstitial and glomerular cells. Notably, in mouse and human tissues, most of the Glce protein was expressed in the kidney tubular cells rather than in the glomerular cells (Figure S2A, Supporting Information). To elucidate the role of *Glce* in kidney fibrosis and injury, we deleted *Glce* from kidney tubules identified by tail genotyping (Figure S2D, Supporting Information). Knockdown efficiency was verified by Western blotting (**Figure** **2**E) and immunofluorescence staining. Compared with the Cdh16/*Glce^flox/flox^* (*Glce* ^‐/‐^) mice, control [Wild type (WT), WT/*Glce^flox/flox^*] littermate mice showed no differences in α‐SMA protein expression (Figure S2E,F, Supporting Information) and maintained a high kidney weight‐to‐body weight ratio at 8 weeks of age, indicating that *Glce* deficiency in tubular cells led to significant retardation of kidney growth (Figure S2G, Supporting Information). Additionally, we found that after UUO surgery, some *Glce* ^‐/‐^ mice exhibited abnormal morphology in the contralateral kidney (Figure 2A). We then measured the Scr and BUN levels to evaluate renal dysfunction. Compared with the control, *Glce* ^‐/‐^ mice exhibited significantly higher levels of Scr and BUN, indicating that *Glce* knockdown induced more severe renal damage (Figure 2B). Histological analysis revealed notable epithelial atrophy, dilated tubules, and interstitial fibrosis in the UUO group. *Glce* knockout mice showed a marked exacerbation of UUO‐induced tubular dilation, atrophy, and notable accumulation of ECM (Figure 2C). *Glce* ablation in tubular cells resulted in higher levels of fibrosis markers in terms of mRNA and protein expression compared with those in WT mice post‐UUO (Figure 2D–F). Next, we overexpressed *Glce* in Cdh16/*Glce^flox/flox^* mouse kidneys in vivo using AAV vectors, followed by UUO or Sham treatment after 6 weeks and were sacrificed at 14 days post‐UUO (Figure S2H, Supporting Information). We first verified that the Glce protein was successfully expressed in mouse kidneys using immunofluorescence analysis (Figure S2I, Supporting Information). Notably, *Glce^‐/‐^* mice showed protection against renal fibrosis, as evidenced by improved kidney morphology and decreased Scr and BUN levels with UUO surgery after the AAV injection. (Figure 2G,H). Furthermore, histological staining revealed that the AAV‐m*Glce* group had a more normal appearance with less collagen fiber deposition in the kidney tissue (Figure 2I). Further investigation showed that *Glce* overexpression prevented higher levels of fibrosis markers compared with the empty vector group (AAV‐ZsG1) after UUO treatment (Figure 2J–L).

### 2.3 Tubule‐Specific *Glce* Deletion Exacerbated EMT Progress via the TGF‐β Signaling Pathway

**Figure 3:**
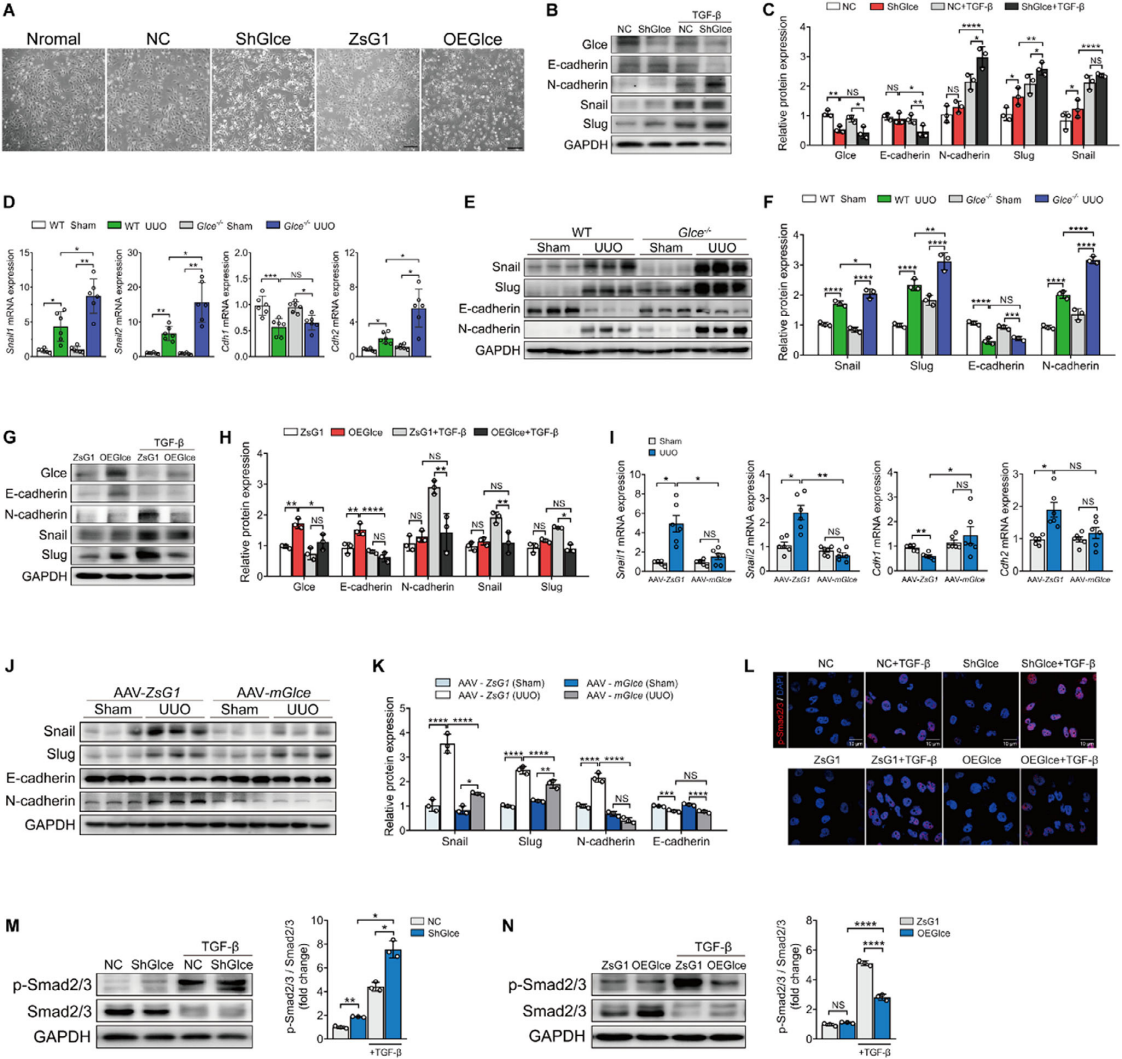
Tubule‐specific *Glce* deletion exacerbated EMT progress via the TGF‐β signaling pathway. A) Morphological changes in HK‐2 cells upon incubation in serum‐free medium (normal) or stable transfection with empty vector (NC), *Glce* shRNA (Sh*Glce*), overexpression vector (ZsG1), or *Glce* overexpression (OE*Glce*). Scale bar: 100 µm. B) HK‐2 cells were incubated separately with either serum‐free medium or 10 ng/mL TGF‐β1. Representative Western blots showing the levels of *Glce*, E‐cadherin, N‐cadherin, Slug, and Snail in HK‐2 cells and C) their corresponding quantification (*n* = 3). D) Relative mRNA expression levels of *Snail1, Snail2, Cdh1, and Cdh2* in WT and *Glce* ^‐/‐^ mice after sham or UUO treatment (*n* = 6). E) Protein expression levels of EMT markers in WT and *Glce* ^‐/‐^ mice after sham or UUO treatment, and F) their corresponding quantification (*n* = 3). G) HK‐2 cells were incubated with or without 10 ng mL^−1^ TGF‐β1. Representative Western blots showing the levels of Glce, E‐cadherin, N‐cadherin, Slug, and Snail in HK‐2 cells and H) their corresponding quantification (*n* = 3). I) mRNA and J,K) protein levels of EMT markers in AAV‐ZsG1 and AAV‐*mGlce* mice after sham or UUO treatment (*n* = 6). L) Immunofluorescence analysis of p‐Smad2/3 (red) in HK‐2 cells with or without 10 ng mL^−1^ TGF‐β1. Scale bar: 10 µm. M,N) Representative Western blots showing the levels of p‐Smad2/3/Smad2/3 in HK‐2 cells and their corresponding quantification (*n* = 3). Data are presented as mean ± SEM. ^*^ *P* < 0.05; ^**^ *P *< 0.01; ^***^ *P* < 0.001; ^****^ *P* < 0.0001 by one‐way ANOVA with Tukey or Dunnett's *post hoc* tests (C,D,F,H,I,K,M, and N).

To determine the role of *Glce* in renal fibrosis resistance, short hairpin RNA against *Glce* (sh*Glce* group) was used to disrupt *Glce* expression in HK‐2 cells, with a control vector as a negative control (NC group). Notably, HK‐2 cells changed from a cobblestone‐like appearance to an elongated fibroblast‐like shape upon *Glce* knockdown, while *Glce* overexpression had no effect on HK‐2 cells (**Figure** **3**A). Additionally, we observed the reduced mRNA and protein levels of *Glce* with increasing induction time by TGF‐β treatment (Figure S3A,B, Supporting Information). This suggests that *Glce* may affect the progression of EMT in renal tubular epithelial cells.^[^ ^16^, ^17^, ^18^ ^]^ Western blot analysis confirmed that *Glce* knockdown in HK‐2 cells significantly increased EMT marker gene expression at the protein level, including N‐cadherin, Snail, and Slug while decreasing E‐cadherin^[^ ^19^ ^]^ (Figure 3B,C). Moreover, the upregulation of EMT markers (*Snail1*, *Snail2*, and *Cdh2*) significantly increased at the mRNA level in *Glce^‐/‐^* mouse kidneys compared with that in the WT group, with no difference in *Cdh1* between WT and *Glce^‐/‐^* mice after UUO (Figure 3D). Immunoblotting of whole‐kidney tissue lysates showed increased protein levels of EMT markers in *Glce^‐/‐^* mice compared with those in WT mice following UUO. The expression of the differentiation marker E‐cadherin decreased in the kidney tissues of WT and *Glce^‐/‐^* mice following UUO (Figure 3E,F). To confirm that *Glce* participates in the renal tubular EMT, we overexpressed *Glce* in HK‐2 cells. The results showed that *Glce* overexpression significantly attenuated TGF‐β1‐induced protein and upregulated Snail, Slug, and N‐cadherin levels in HK‐2 cells (Figure 3G,H). Additionally, the results showed that overexpression of *Glce* resulted in marked downregulation of *Snail1*, *Snail2*, and *Cdh2*, alongside increased *Cdh1* compared with the control group post‐UUO (Figure 3I). Consistent with these results, immunoblotting showed a marked decrease in Snail, Slug, and N‐cadherin levels in the AAV‐m*Glce* group post‐UUO (Figure 3J,K). Collectively, these results demonstrate that *Glce* is critical for protection in response to kidney injury.

Therefore, we speculated that *Glce* mediates its differentiation effects by regulating downstream signaling effectors of TGF‐β1. We examined the activities of the TGF‐β‐Smad2/3 pathway, known to play a critical role in kidney fibrosis progression.^[^ ^20^, ^21^ ^]^ Western blot analysis showed that tubule‐specific *Glce* deletion promoted Smad2/3 phosphorylation, which could be reversed by *Glce* overexpression (Figure 3M,N). Similar results were obtained from the immunofluorescence images (Figure 3L). Since TGF‐β1‐mediated Smad2/3 activation is strictly dependent on transmembrane TGF‐β receptor type I (TGFBR1), we designed an experimental procedure and treated animals with the TGFBR1 inhibitor (SB431542) (Figure S3C, Supporting Information). Notably, in SB431542‐treated SD mice, kidney function indices (Scr and BUN) improved post‐UUO, whereas no significant change was observed in WT mice. (Figure S3D, Supporting Information). Additionally, collagen deposition areas and tubular necrosis became smaller in the *Glce* ^‐/‐^ mice after treatment with SB431542 post‐UUO, while the WT mice showed no signs of improvement, with some even worsening compared with *Glce* ^‐/‐^ mice (Figure S3E, Supporting Information). Immunohistochemistry results showed that protein expression of α‐SMA and COL1 decreased in the kidneys of *Glce* ^‐/‐^ mice post‐UUO, while no significant changes were found in WT mice (Figure S3F, Supporting Information). Western blotting indicated that SB431542 treatment suppressed Smad2/3 phosphorylation and reduced N‐cadherin, Snail, and Slug levels in both groups (Figure S3H,I, Supporting Information). As the disease progressed, the obstructed kidneys of WT and *Glce* ^‐/‐^ mice maintained lower *Cdh1* expression levels and higher *Snail1*, *Snail2*, and *Cdh2* genes compared with the control mice, and treatment with SB431542 reversed this trend in *Glce* ^‐/‐^ mice, but had no effect on *Snail2* and *Cdh1* in WT mice (Figure S3G, Supporting Information).

### 2.4 *Glce* Binds to EGF Receptor to Suppress its Activation and Impact on Renal Fibrosis

**Figure 4:**
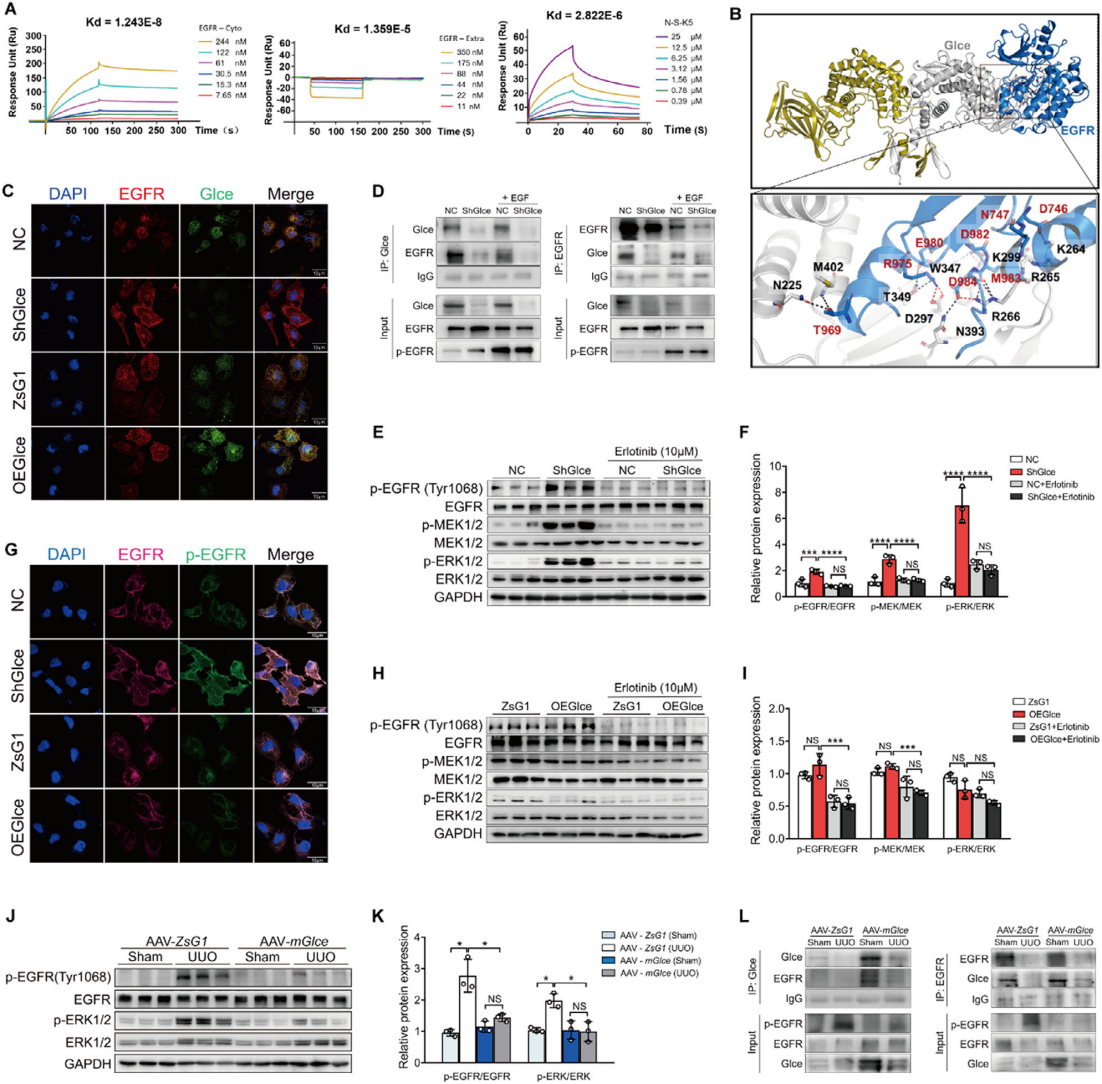
Glce binds to the EGF receptor to suppress its activation and impact on renal fibrosis. A) The surface plasmon resonance (SPR) test revealed direct binding between Glce and EGFR proteins. Sensorgrams of intracellular EGFR protein (7.65–244 µm) binding to Glce protein (left), extracellular EGF receptor protein (11–350 µm) binding to the Glce protein (middle), positive control polysaccharide N‐S‐K5 (0.39–25 µm) binding to Glce (right). B) The docking model of the Glce‐EGFR complex. The intracellular EGFR domain is shown in blue. The two monomers of h*Glce* are shown in gold and silver. The binding interface is marked with a black rectangular box and a close‐up view of the interactions is shown in the bottom panel. The key residues involved in Glce (black) and EGFR (red) interactions are shown as sticks and are colored silver and blue, respectively. The salt bridges and hydrogen bonds are indicated by red and black dashed lines, respectively. C) Immunofluorescent co‐localization analysis of Glce (green) and EGFR (red) in HK‐2 cells transfected with different plasmids. Scale bar: 10 µm. D) Co‐immunoprecipitation (Co‐IP) analysis of interactions between Glce and EGFR in HK‐2 cells. GAPDH was used as the input loading control. Data are representative of at least two independent experiments. E) Representative Western blots of p‐EGFR (Tyr1068), EGFR, p‐MEK1/2, MEK1/2, p‐ERK1/2, and ERK1/2 in HK‐2 cells transfected with sh*Glce* and vector control (NC) followed by Erlotinib (10 µm) and F) protein quantification (*n* = 3). G) Immunofluorescent co‐localization analysis of p‐EGFR (green) and EGFR (rose) in HK‐2 cells transfected with different plasmids. Scale bar: 10 µm. H) Representative Western blots of p‐EGFR (Tyr1068), EGFR, p‐MEK1/2, MEK1/2, p‐ERK1/2, and ERK1/2 in HK‐2 cells transfected with *Glce*‐overexpression plasmid and vector control (ZsG1) followed by Erlotinib (10 µm) and I) protein quantification (*n* = 3). GAPDH was used as a loading control. J) Representative Western blots of p‐EGFR (Tyr1068), EGFR, p‐ERK1/2, ERK1/2, and K) protein quantification in the kidneys of *Glce* ^‐/‐^ mice subjected to sham or UUO treatment after AAV‐encoding full‐length *Glce* injection (*n* = 3). L) Co‐IP analysis of the interactions between Glce and EGFR in the kidneys of *Glce* ^‐/‐^ mice with sham or UUO treatment after AAV injection. GAPDH was used as the input loading control. Data are representative of at least two independent experiments. Data are presented as mean ± SEM. ^*^ *P* < 0.05; ^**^ *P *< 0.01; ^***^ *P* < 0.001; ^****^ *P* < 0.0001 by one‐way ANOVA with Tukey or Dunnett's *post hoc* tests (F,I, and K).

Although *Glce* is crucial in regulating the TGFβ1/Smad pathway, no direct interaction between *Glce* and TGFBR1 was observed upon further evaluation (data not shown). Reportedly, enhanced activation of the EGF receptor (EGFR) is associated with the development and progression of renal fibrosis, and genetic or pharmacological blockade of EGFR can inhibit this condition.^[^ ^22^ ^]^ To explore whether EGFR might be a target of Glce protein, we conducted a surface plasmon resonance (SPR) experiment and found that Glce protein had a strong binding interaction with the intracellular domain of EGFR in vitro but did not bind to its extracellular domain. N‐sulfated K5 polysaccharide, a classical substrate^[^ ^23^ ^]^ for the *Glce* enzyme, was used as a positive control (**Figure** **4**A). To further confirm these results and analyze the binding details, a docking analysis was performed. As shown in Figure 4B (Top panel), the cytoplasmic domain of EGFR is predicted to bind to a deep hydrophobic groove of Glce protein that is spanned by (α/α)4‐barrel domain and the β‐sandwich domain. The majority of the buried surface area in the docking Glce‐EGFR complex was calculated as 2363 Å2. The structure indicated by the dashed lines serves as the predicted site for key interactions (Lower panel). Glce protein potentially forms 11 hydrogen bonds with EGFR (black dashed lines), including N255‐T969’, M402‐T969’, T349‐E980’, T349‐R975’, N393‐D984’, R266‐D984’, W347‐D982’, S265‐N747’, and R266‐M983’, alongside salt bridges between 4 residue pairs, including D297‐R975’, R266‐D984’, K264‐D746’, and K299‐D982’ (red dashed lines). Detailed distance information is provided in Supplemental Table S1. Next, we speculated that Glce co‐localizes with EGFR in renal tubular cells (Figure 4C). Glce displayed strong co‐localization with EGFR in each group, except for the Sh*Glce* group in HK‐2 cells. To confirm these interactions, we performed co‐immunoprecipitation (Co‐IP) assays. Although the precipitate confirmed the binding of Glce to EGFR in HK‐2 cells, the interaction between Glce and EGFR markedly decreased upon EGFR activation by EGF (Figure 4D). Thus, we speculated that *Glce* may be involved in the activation of EGFR. To investigate whether *Glce* regulates EGFR phosphorylation, we analyzed the major phosphorylation sites of EGFR at Tyr1045, Tyr1068, Tyr1148, and Tyr1173/Tyr1248 by immunoblotting. Immunoblotting demonstrated that *Glce* knockdown increased EGFR phosphorylation at Tyr1068 but had no measurable effect on other tyrosine phosphorylation sites (Figure S4A, Supporting Information). Immunofluorescence staining showed an increased level of phosphorylated EGFR in the Sh*Glce* group than in the other groups (Figure 4G). Activation of the EGFR/MAPK pathway leads to renal fibrosis progression.^[^ ^24^ ^]^ To study the effect of *Glce* on the MAPK pathway, we assessed the activation of this pathway in *Glce* knockdown cell lines. The results showed that *Glce* knockdown did not significantly alter the expression of p‐p38 and p‐JNK but markedly activated ERK1/2 phosphorylation and its downstream signaling pathways (Figure 4E,F; Figure S4B,C, Supporting Information). We then overexpressed *Glce* and detected the MAPK pathway expression; however, no significant change was observed compared with the vector control group (Figure 4H,I). These results confirmed that *Glce* knockdown might activate the EGFR/ERK pathways; however, *Glce* overexpression did not affect EGFR phosphorylation in HK‐2 cells. Immunoblotting of whole‐kidney tissue lysates showed reduced protein levels of p‐EGFR(Tyr1068) and p‐ERK1/2 in AAV‐m*Glce* mice compared with those in AAV‐ZsG1 mice post‐UUO (Figure 4J,K). Additionally, protein Co‐IP experiments in AAV‐m*Glce* mouse kidneys showed physical binding between *Glce* and EGFR, whereas this interaction decreased in UUO‐induced fibrotic kidneys (Figure 4L).

### 2.5 Inhibition of the EGFR Signaling Pathway Ameliorated TGF‐β/Smad Cascade and Renal Fibrosis in *Glce^‐/‐^* and WT Mice

**Figure 5:**
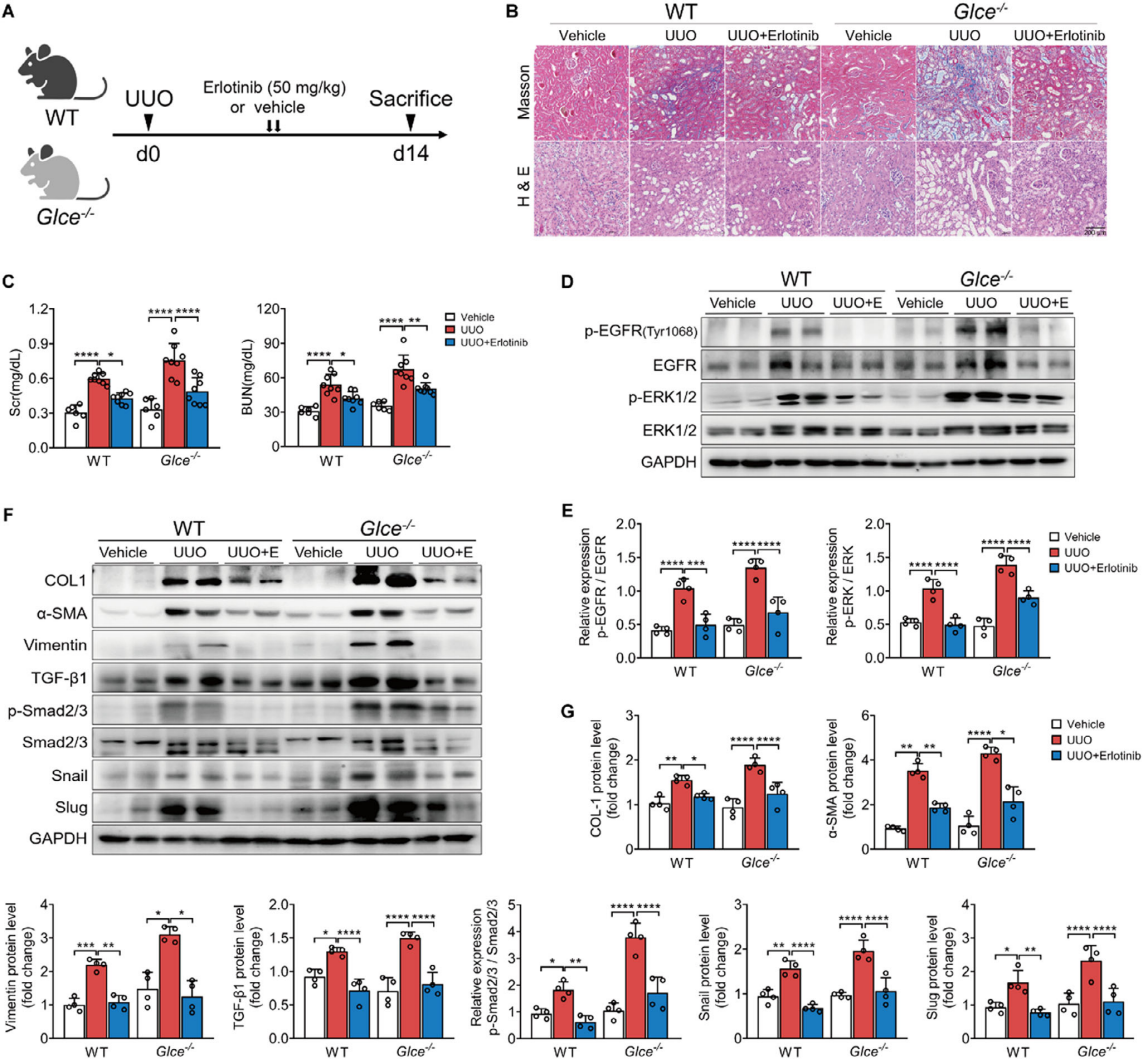
Inhibition of EGFR signaling pathway ameliorated TGF‐β/Smad cascade and renal fibrosis in *Glce^‐/‐^* and WT mice. A) Schematic of erlotinib treatment in the UUO‐induced mice. B) Representative H&E and Masson staining of WT and *Glce* ^‐/‐^ mouse kidneys following erlotinib treatment 14 days post‐UUO. Scale bar: 100 µm. C) Scr and BUN levels in WT and *Glce* ^‐/‐^ mice following erlotinib treatment 14 days after UUO. Sham (*n* = 6), UUO (*n* = 8), and UUO + Erlotinib groups (*n* = 8). D,E) Western blot analysis of p‐EGFR (Thr1068), EGFR, p‐ERK1/2, ERK1/2, and GAPDH in whole‐kidney lysates from WT and *Glce* ^‐/‐^ mice 14 days after UUO, and the corresponding quantification of p‐EGFR/EGFR and p‐ERK1/2/ERK1/2 (*n* = 4). F,G) Representative Western blots of Collagen 1, α‐SMA, Vimentin, TGF‐β1, p‐Smad2/3, Smad2/3, Snail, Slug and protein quantification in WT and *Glce* ^‐/‐^ mouse kidneys following erlotinib treatment 14 days after UUO (*n* = 4). Data are representative of at least three independent experiments. Data are presented as mean ± SEM. ^*^ *P* < 0.05; ^**^ *P *< 0.01; ^***^ *P* < 0.001; ^****^ *P* < 0.0001 by one‐way ANOVA with Tukey or Dunnett's *post hoc* tests (C,E, and G).

TGF‐β‐mediated tissue fibrosis relies on a persistent feed‐forward mechanism of EGFR/ERK activation.^[^ ^25^ ^]^ To explore the effects of *Glce* in the pathogenesis and progression of renal fibrosis in vivo through the EGFR/ERK signaling pathway, the EGFR inhibitor, erlotinib, was administered post‐UUO (**Figure** **5**A). Histological analysis indicated significant epithelial atrophy, dilated tubules, and interstitial fibrosis post‐UUO, of which *Glce* ^‐/‐^ mice exhibited more severe changes. After 14 days of erlotinib administration, renal damage significantly improved (Figure 5B). Additionally, biochemical assays demonstrated that Scr and BUN levels increased by varying degrees in UUO‐treated animals; however, the levels significantly decreased in both erlotinib‐treated groups (Figure 5C). Western blot analysis of kidney lysates confirmed that the protein levels of p‐EGFR(Tyr1068) and p‐ERK1/2 increased in WT and *Glce* ^‐/‐^ mice post‐UUO, which was attenuated by erlotinib treatment (Figure 5D,E). As shown in Figure 5F, the protein levels of COL1, α‐SMA, and vimentin increased post‐UUO, whereas erlotinib administration led to a marked reduction of these proteins in WT and *Glce^‐/‐^* mice. Compared with WT mice, UUO‐treated *Glce* ^‐/‐^ mice showed increased protein expression. Enhanced expression of TGF‐β1, p‐Smad2/3, and its downstream EMT markers significantly decreased after administration of erlotinib (Figure 5G). Therefore, *Glce* deletion activated the EGFR signaling pathway. Aberrant EGFR activation resulted in increased expression of TGF‐β1 and then exacerbated the EMT process via SMAD‐dependent pathways in kidneys.

### 2.6 AAV Encoding Mutant *Glce* Orthotopically Injected into *Glce^‐/‐^* Mice can Ameliorate Renal Fibrosis

**Figure 6:**
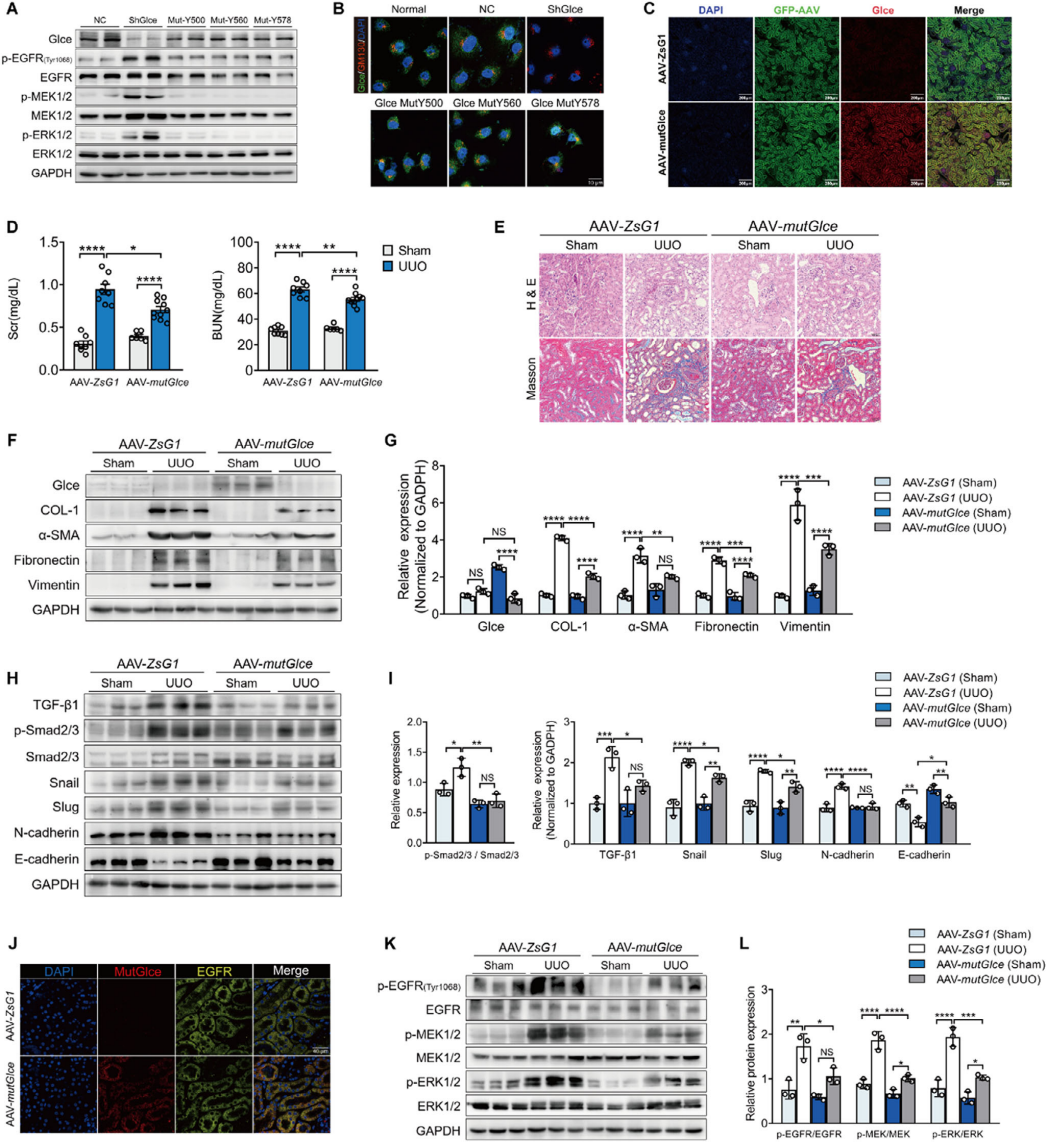
AAV encoding mutant *Glce* orthotopically injected to *Glce* ^‐/‐^ mice ameliorated renal fibrosis. A) Representative Western blots of *Glce*, p‐EGFR (Thr1068), EGFR, p‐MEK1/2, MEK1/2, p‐ERK1/2, and ERK1/2 in HK‐2 cells transfected with plasmids encoding control vector (NC), sh*Glce*, and mutant *Glce* (Y500F, Y560F, and Y578F, respectively). GAPDH was used as the loading control, and the data are representative of two independent experiments. B) Immunofluorescence staining of Glce (green) and GM130 (red) in HK‐2 cells of different genotypes. Scale bar: 10 µm. C) Immunofluorescence staining of Glce (red) and GFP (green) in mice 6 weeks after AAV encoding full‐length mutant *Glce* injection. Scale bar: 200 µm. (D) Scr and BUN levels of *Glce* ^‐/‐^ mice subjected to sham or UUO treatment after AAV encoding full‐length mutant *Glce* injection. AAV‐ZsG1 Sham (*n* = 8), UUO (*n* = 8), AAV‐m*Glce* Sham (*n* = 6), and UUO (*n* = 10) groups. (E) Representative H&E and Masson staining of *Glce^‐/‐^* mouse kidneys with sham or UUO treatment 6 weeks after AAV‐ZsG1 or AAV‐mut*Glce* injection. Scale bar: 100 µm. (F, G) Representative Western blots of Glce, Collagen 1, α‐SMA, Fibronectin, Vimentin, and protein quantification in the kidneys of *Glce* ^‐/‐^ mice with sham or UUO treatment after AAV‐ZsG1 or AAV‐mut*Glce* injection (*n* = 3). H,I) Representative Western blots of TGF‐β1, p‐Smad2/3, Smad2/3, Snail, Slug, E‐cadherin, N‐cadherin, and protein quantification in the kidneys of *Glce* ^‐/‐^ mice with sham or UUO treatment after AAV injection (*n* = 3). J) Immunofluorescence co‐localization analysis of mutant Glce (red) and EGFR (yellow) in the kidneys of *Glce* ^‐/‐^ mice after AAV‐ZsG1 or AAV‐mut*Glce* injection. Scale bar: 20 µm. K,L) Representative Western blots of p‐EGFR (Thr1068), EGFR, p‐MEK1/2, MEK1/2, p‐ERK1/2, and ERK1/2 in the kidneys of *Glce* ^‐/‐^ mice subjected to sham or UUO treatment after AAV injection (*n* = 3). Data are representative of at least three independent experiments unless indicated otherwise. Data are presented as mean ± SEM. ^*^ *P* < 0.05; ^**^ *P *< 0.01; ^***^ *P* < 0.001; ^****^ *P* < 0.0001 by one‐way ANOVA with Tukey or Dunnett's *post hoc* tests (D, G, I and L).

According to SPR results, *Glce* may strongly interact with the intracellular region of EGFR. Molecular docking analysis revealed that the potential EGFR binding regions in human Glce protein are mostly located within its classical β‐sandwich domain, excluding its classical enzyme active sites. Therefore, to determine the necessity of the enzymatic function of *Glce* in EGFR activation and TGF‐β1‐induced EMT during renal fibrosis pathogenesis, we selected three critical sites for the enzyme activity including, Y500, Y560, and Y578, for mutational studies.^[^ ^12^ ^]^ HK‐2 cells with stable *Glce* knockdown were transfected with plasmids containing mutant genes. The protein expression levels of p‐EGFR, p‐MEK1/2, and p‐ERK1/2 were markedly higher in *Glce* knockdown HK‐2 cells, whereas mutant *Glce* showed no effect on the EGFR/ERK pathway (**Figure** **6**A). Previous studies suggested that HS is biosynthesized in the Golgi network, where the polysaccharide chain is polymerized and modified by a series of Golgi‐located enzymes.^[^ ^26^ ^]^ Notably, we found that the mutant *Glce* was expressed successfully and distributed in the cytoplasm of renal tubular cells, besides the Golgi (Figure 6B). To explore the therapeutic potential of mutant *Glce*, we constructed AAV vectors encoding GFP and all three mutant sites (AAV‐mut*Glce*) simultaneously and injected them orthotopically into the kidneys of *Glce* ^‐/‐^ mice, using an empty vector plasmid (AAV‐ZsG1) as a control. Immunofluorescence staining showed that mutant *Glce* was successfully overexpressed in renal tubular epithelial cells (Figure 6C). Compared with those injected with AAV‐ZsG1, the overexpression of mutant *Glce* (AAV‐ mut*Glce*) resulted in lower levels of Scr and BUN following UUO (Figure 6D). Overexpression of mutant *Glce* reduced the extent of renal fibrosis compared with AAVZsG1‐treated mice (Figure 6E). Immunoblotting showed reduced fibrosis protein levels in AAV‐mut*Glce* mice compared with AAVZsG1 mice following UUO (Figure 6F,G). Additionally, expression of TGF‐β1 and EMT markers were markedly lower in AAV‐ mut*Glce* mice compared with AAV‐ZsG1 mice subjected to UUO (Figure 6H,I). Previous studies indicated that UUO induced tubular epithelial autophagy but failed to trigger EMT,^[^ ^27^, ^28^ ^]^ whose clinical relevance remained controversial due to challenges in demonstrating it in vivo. In light of this, we sought to determine whether *Glce* attenuated renal fibrosis by modulating tubular cell autophagy and our experiments indeed supported this hypothesis. Immunofluorescence analysis revealed elevated LC3‐positive puncta and reduced SQSTM1 expression in renal tissues of tubule‐specific *Glce* knockout mice compared with wild‐type group (Figure S5A, Supporting Information). This finding was corroborated in cellular experiments. This indicated that the protective effect of *Glce* on renal tubules is a more complex and comprehensive regulatory mechanism, and autophagy might only be one aspect of it (Figure S5B–E, Supporting Information). However, drawing on the latest experimental evidence presented in this study, we further emphasized that *Glce* regulated EMT at least partially contribute to the promotion of fibroblast activation (Figure S6A–D, Supporting Information), thereby enhancing the accuracy and rigor of our findings. Similarly, Western blot analysis revealed that mutant *Glce* overexpression induced a marked decrease in the EGFR/ERK signaling pathway phosphorylation compared with vector control mice in UUO‐treated kidneys (Figure 6K,L). Furthermore, immunofluorescence analysis showed co‐localization of EGFR and mutant *Glce* in kidney tissues from AAV‐mut*Glce* mice (Figure 6J). Collectively, AAV‐ mut*Glce* mice subjected to UUO showed better tubular health, renal function, and less interstitial fibrosis.

## 3 Discussion

Kidney fibrosis is a hallmark of all forms of CKD. Given the complexity of fibrosis involving different mechanisms and factors, clinical therapies for renal fibrosis are quite limited.^[^ ^29^ ^]^ *Glce* is a highly evolutionarily conserved enzyme expressed in various organs in the body.^[^ ^30^ ^]^ Hence, we constructed renal tubule‐specific *Glce* knockout mice and observed their phenotypes during development. Notably, these mice did not exhibit embryonic lethality and showed no significant differences in activity and appearance compared with the control mice. However, renal weight/body weight ratio measurements after sacrifice confirmed renal growth retardation in *Glce* ^‐/‐^ mice, with some displaying a unilateral absence of kidneys. We found that *Glce* levels were lower in patients with various types of CKD and negatively correlated with the degree of fibrosis at different stages. This suggests that *Glce* may be associated with renal fibrosis. We examined the typical fibrosis signaling pathways, including TGF‐β/Smad, Wnt/β‐catenin, Hedgehog, PI3K/Akt/mTOR, and Notch in the *Glce* knockout mice.^[^ ^31^, ^32^, ^33^, ^34^, ^35^ ^]^ However, the result indicated that except for TGF‐β/Smad, *Glce* knockout did not induce change in these signaling pathways compared with WT mice.

Our findings suggest that *Glce* is not a direct cause of fibrosis; rather, *Glce* knockdown promotes EMT in HK‐2 cells. TGF‐β1, a key EMT mediator, induces transcription of several mesenchymal genes and increases the activity of EMT transcription factors via SMAD factors in renal epithelial cells.^[^ ^16^, ^36^ ^]^ Recent studies have established a consensus on several critical factors involved in the development and progression of kidney fibrosis, including cell cycle arrest, defective cellular metabolism, and EMT.^[^ ^37^ ^]^ Renal tubular cells play critical roles in fibrosis, leading to suggestions that targeting the aberrant expression of specific genes such as snail1, DsbA‐L, and Tfam in tubular cells might directly or indirectly ameliorate kidney dysfunction.^[^ ^38^, ^39^, ^40^ ^]^ Iwano reported that fibroblasts originating from epithelial‐to‐mesenchymal transition accounted for ≈36% of FSP1⁺ fibroblasts in the context of renal fibrosis.^[^ ^41^ ^]^ However, the function of EMT against kidney fibrosis was controversial. In contrast, more recent study found that only ≈5% of myofibroblasts in kidney fibrosis were derived from EMT.^[^ ^42^ ^]^ The subsequent studies demonstrated that injured tubular epithelial cells (TECs) undergone a partial EMT to acquire some of the mesenchymal characteristics instead of converting to a complete fibroblastic phenotype. The contribution of complete EMT to the pool of interstitial myofibroblasts appears to be minimal,^[^ ^42^ ^]^ whereas partial EMT has garnered greater attention due to its potential functional significance.^[^ ^43^, ^44^, ^45^ ^]^ Partial EMT is a state in which injured renal tubular epithelial cells co‐express epithelial and mesenchymal markers while remaining within tubules, accompanied by G2/M cell cycle arrest. This leads to impaired regeneration, dysfunctional repair, and an altered secretome. Initially triggered as a protective stress response, partial EMT ultimately drives TECs toward a secretory phenotype, enabling persistent release of pathological mediators that activate myofibroblast precursors. Since the majority of the kidney parenchyma is comprised of tubular epithelium, this insight emphasizes the potential for focusing on renal tubular cells to advance further clinical applications in CKD treatment.

Our study revealed that *Glce* deficiency in renal tubular cells promoted TGF‐β/Smad pathway activation predominantly associated with TGFBR1 rather than Smad3, however, inhibition of TGF‐β/Smad pathways partially relieved renal fibrosis in *Glce* ^‐/‐^ mice after UUO. Further investigation indicated that Glce binds to neither TGF‐β1 nor TGFB1 receptor but strongly binds to EGFR, indicating that *Glce* overexpression prevents the abnormal activation of the EGFR pathway and improves renal tubulointerstitial fibrosis. EGFR is a transmembrane glycoprotein comprising an extracellular region, transmembrane domain, and intracellular tyrosine kinase domain.^[^ ^46^ ^]^ Using SPR, we observed that Glce binds with high affinity to the intracellular domain of EGFR, suggesting their binding may primarily influence EGFR kinase domain activation. This is supported by docking predictions, which are consistent with the results observed in our in vitro and in vivo experiments. Thus, we believe that the Glce‐EGFR binding effects act as an ‘on‐off’ switch to regulate the activation of EGFR in the kidneys.

Evidence suggests that HS plays a pivotal role in various pathological fibrotic conditions.^[^ ^47^ ^]^ Therefore, we speculated that the renoprotective effect of *Glce* relies on its function in catalyzing HS formation. However, the results indicated that the *Glce* enzyme mutation in tubular cells did not activate the EGFR pathway but still ameliorated renal fibrosis in the UUO‐induced *Glce* ^‐/‐^ mice. Additionally, we observed that Glce was distributed near the cellular membrane and co‐localized with EGFR, in addition to the Golgi. Although most enzymes modulate fibrosis through their catalytic mechanisms, non‐enzymatic functions of proteins also contribute to fibrosis progression. Recently, we showed that Glce protein may bind to GDF15 to maintain the energy metabolism balance in a non‐enzymatic manner.^[^ ^13^ ^]^ Thus, we infer that, in addition to playing an epimerase function in the Golgi apparatus, *Glce* can regulate EGFR, activating distinct signaling pathways via a non‐enzymatic mechanism. Overall, the protective effects of *Glce* against renal fibrosis suggest its multifunctional roles, revealing a novel function for *Glce*.

In conclusion, this study is the first to demonstrate the important role of *Glce* in the maintenance of kidney function and renal fibrosis. We found that *Glce* is bound to the intracellular domains of EGFR in normal tubular cells. Tubule‐specific *Glce* loss causes the activation of EGFR signaling and the TGF‐β‐Smad cascade, leading to more severe tubulointerstitial injury and fibrosis after UUO. This research, however, is subject to several limitations. For instance, in our murine in vivo model studies, the renal tissue specimens collected encompassed nearly the entire renal tissue. This sampling strategy does not fully eliminate potential confounding effects from interstitial fibroblasts, immune cells, or vascular endothelial cells. To precisely delineate the role of *Glce* in renal pathophysiology, in the future, we will focus on the functions of *Glce* in more types of kidney cells. Collectively, this study reveals a novel nephroprotective effect of *Glce*, distinguishing it from its currently known enzymatic function and providing new potential targets for the treatment of CKD.

## 4 Experimental Section

### Study Design

To investigate the protective role of renal *Glce* in the pathogenesis of kidney fibrosis was aimed. Using IHC staining, a reduction in *Glce* in renal biopsies from patients with different types of CKD compared with those with MCD was identified, which is closely related to the degree of fibrosis deterioration. Therefore, it was hypothesized that *Glce* plays an important role in kidney fibrosis progression. To test this, UUO and FA‐treated mice as models of CKD were used. Using the Cdh16/*Glce^flox/flox^* (*Glce^‐/‐^*) mouse model, the effect of *Glce* on renal function was evaluated. RT‐PCR and Western blot analyses were conducted, demonstrating that tubule‐specific *Glce* deletion exacerbates EMT via the TGF‐β signaling pathway using HK‐2 cells.

To explore the molecular mechanisms underlying renal fibrosis caused by *Glce* deletion, SPR and Co‐IP assays was performed to assess the potential interaction of Glce with EGFR. To further confirm the results and analyze the binding details, a molecular model of the interaction sites for Glce protein binding to EGFR was constructed by docking analysis. To further determine whether Glce co‐localizes with EGFR in renal tubular cells, a confocal immunofluorescence analysis was performed. To study the effect of *Glce* on the MAPK pathway, the activation of this pathway was assessed in *Glce* knockdown and overexpression cell lines. Additionally, to explore the effects of *Glce* on the pathogenesis and progression of renal fibrosis in vivo through the EGFR/ERK signaling pathway, the EGFR inhibitor, erlotinib, was administered following UUO surgery. To determine whether the enzymatic function of *Glce* was essential for EGFR activation and TGF‐β1‐Smad cascade in the pathogenesis of renal fibrosis, three sites that were considered crucial for the enzyme activity was selected, including Y500, Y560, and Y578, for mutational studies. To understand the therapeutic potential of mutant *Glce*, AAV vectors encoding GFP and all three mutant sites (AAV‐mut*Glce*) simultaneously were constructed and injected them orthotopically into the kidneys of *Glce^‐/‐^* mice. An empty vector plasmid (AAV‐ZsG1) was used as a control. Within littermate groups, animals were randomly selected from each experimental group, with biological replicates indicated in the figure legends.

### Human Renal Biopsy Samples

Patients were recruited from the Department of Nephrology at the Shanghai Changzheng Hospital in Shanghai. All clinical records used in this study were approved by the Institutional Review Board. The raw data used in this study were obtained from the clinical records of the participating patients and were included in a database after anonymization (2022SL064). Patients were grouped into MCD, IgA, LN, MN, and DN according to the different features of the disease. Several trained nephrologists evaluated clinical imaging to define specific pathological types of renal disease. Blood biochemical parameters were extracted from the custom system of Shanghai Changzheng Hospital.

### Mouse Models

All animals were maintained in the core animal facility, and the study was approved by the Institutional Animal Care and Use Committee of the Shanghai Institute of Materia Medica (*IACUC* no. 2019‐06‐DK‐79; 2019‐06‐DK‐80). Eight‐ to ten‐week‐old C57BL/6 mice were obtained from the Shanghai SLAC Laboratory Animal Company (Shanghai, China). All experiments were conducted on group‐housed animals (4–5 animals per cage) under a 12‐h light/dark cycle with free access to food and water.

Floxed *Glce* mice and Cdh16‐cre transgenic mice were purchased from the Model Animal Research Center of Nanjing University (Nanjing, China) and backcrossed with C57BL/6 mice for >8 generations to produce congenic strains. C57BL/6 *Glce ^flox/flox^* mice were crossed with mice expressing Cre recombinase (Cre) to generate renal tubular‐specific *Glce* knockout mice (*Glce* ^‐/‐^ mice). Mice with two WT alleles and Cre expression were defined as WT mice. Genotyping by tail preparation and PCR was performed at two weeks of age, with subsequent experiments carried out when the mice were 8 to 12 weeks old unless otherwise noted.

In the UUO‐induced mouse model, eight‐week‐old male mice were randomly divided into sham operation and UUO groups. Sham‐operated mice served as controls (*n* = 15 per group). For the UUO model, sevoflurane was used to maintain general anesthesia for convenient skin preparation and surgery. After the surgical area was strictly disinfected, the mice underwent ligation of the left ureter and were sacrificed two weeks later.

In the FA‐treated mouse model, eight‐week‐old male mice were randomly divided into control and FA‐treated groups. For the FA model, mice were induced with a single intraperitoneal injection of FA (250 mg kg^−1^, dissolved in 300 mm NaHCO_3_) and sacrificed one month later.

In the SB431542 treatment mouse model, eight‐week‐old male mice were randomly divided into sham, UUO, and UUO+SB431542 groups. Sham and UUO groups were subjected to the processes described above. In the UUO+SB431542 group, mice were treated with continuous intraperitoneal injections of SB431542 (2 mg kg day^−1^, dissolved in 10% PEG400 solution) for 10 days post‐UUO surgery and were sacrificed four days later.

In the Erlotinib‐treated mouse model, eight‐week‐old male mice were randomly divided into sham, UUO, and UUO+Erlotinib groups. Sham and UUO groups were subjected to the aforementioned processes. In the UUO+Erlotinib group, the mice were orally administered erlotinib (50 mg kg day^−1^, suspended in sterile PBS before administration) once daily for 14 consecutive days after surgery.

### Cell Culture and Treatments

HK‐2 cells (Cell Bank, Chinese Academy of Sciences, China) were maintained in DMEM/F12 containing 10% FBS, penicillin (100 units mL^−1^), streptomycin (100 µg mL^−1^), and 1% L‐glutamine unless otherwise noted. To detect the mRNA and protein level of *Glce*, HK‐2 cells (2 × 10^5^/well) were seeded in a 60 mm dish and then stimulated with 10 ng mL^−1^ TGF‐β at different times. In subsequent experiments, HK‐2 cells transfected with various plasmids were stimulated with or without TGF‐β (10 ng mL^−1^) for 24 h. To determine the action target, HK‐2 cells were treated with TGF‐β (10 ng mL^−1^) for 24 h in the presence of SIS3 (5 µm) or SB431542 (10 µm).

### Statistical Analysis

Statistical analyses were performed using GraphPad Prism 8.0 software, with values expressed as the standard error of the mean. Two‐tailed Student's t‐test was used to test for significance between the experimental and control groups. When three or more groups were assessed, one‐way ANOVA with Tukey's or Dunnett's multiple‐comparison *post hoc* test was used. Correlation analyses were performed using Spearman's correlation coefficients. *P* values < 0.05 are considered statistically significant.

Additional details for all methods are provided in the Supplementary Methods.

## Conflict of Interest

The authors declare no conflict of interest.

## Author Contributions

X.J., J.W., and J.N. contributed equally to this work and should be considered as co–first authors. The study was designed by X.J., K.D., J.W., and C.M.; Experimental analysis was performed by X.J. and J.W.; Animal experiments were performed by X.J. and Z.D., which was assisted by L.H.; Human renal immunohistochemical stains and related independent pathological evaluation were performed by J.W. and R.W.; Protein‐protein docking analysis was performed by X.D.; Surface plasmon resonance assay was performed by X.J. and X.W.; X.J., and J.N. wrote the manuscript; K.D. and C.M. performed the critical revision and editing of the manuscript; All authors approved the final version of the manuscript.

## Supporting information



Supporting Information

## Data Availability

The data that support the findings of this study are available from the corresponding author upon reasonable request.
