# Supplementary material for: D‐glucuronyl C5‐Epimerase Binds to EGFR to Suppress Kidney Fibrosis
Source: Adv Sci (Weinh). 2025 Aug 11;12(40):e16216. doi: 10.1002/advs.202416216 (PMC12561193; doi:10.1002/advs.202416216)
Supplement: Supplementary file 1 — Supporting Information [file ADVS-12-e16216-s001.docx]

**Supporting Information**

**D-glucuronyl C5-epimerase binds to EGFR to suppress kidney fibrosis**

***Authors:*** *Xiaoqi Jing^1^**^#^, Jun Wu^2#^, Jingru Ning^3#^, Xiaoyu Ding^4^, Zhenyun Du^1^, Xiaojiang Wang^5^, Lulin Huang^1^, Ran Wang^2^, Changlin Mei^2^*, Kan Ding^1,3,6^**

**Supplementary materials**

**The file includes:**

Supplementary Methods

Fig. S1 to S6

Tables S1 to S3

Supplementary References

**Supplementary methods**

***Immunofluorescence***

The mice’s kidneys were harvested and snap-frozen in optimal cutting temperature compounds (OCT, Fisher). Cryosections at 20 μm were made and used for immunofluorescent staining. Cells or mice kidney sections were fixed (4% PFA) and permeabilized (0.3 % Triton X) before blocking with 5% BSA (37 °C, 1 h), stained with corresponding primary antibodies (1: 200) overnight at 4 °C, washed, and then stained with secondary antibody (37 °C, 1 h; 1: 200), counterstained with DAPI (1 μg/mL) and then analyzed with two-photon confocal microscopy (Leica, SPS). Images were analyzed using ImageJ pro plus.

***Immunohistochemistry***

The paraffin-embedded tissues were dewaxed by xylene and then rehydrated in a graded ethyl alcohol series. After washing the sections, 0.3% hydrogen peroxide solution was applied to block endogenous peroxide for 30 min at room temperature. The sections were blocked with 5% BSA for 30 min and then with primary antibodies diluted in 5% BSA overnight at 4 °C. Subsequently, the slides were incubated with suitable secondary antibodies at room temperature for 1 h and treated with DAB. Immunohistochemistry was performed using the Ventana BenchMark GX autostainer (Roche Diagnostics, Basel, Switzerland).

***Renal function evaluation***

Serum was obtained from humans or mice and then analyzed for levels of creatinine and blood urea nitrogen (BUN) by AU 400 autoanalyzer (Olympus, Japan).

***Histology and Histopathology***

Kidneys were fixed in 10% neutral buffered formalin and embedded in paraffin. 5 μm sections were cut for hematoxylin and eosin (H&E) and Masson’s trichrome (MTS), periodic acid-Schiff (PAS) staining. The extent of renal injury was estimated by morphometric assessment of tubular damage and interstitial fibrosis. Representative images were acquired with a Nikon microscope (Nikon, Tokyo, Japan).

***RNA extraction, RT-PCR, and quantitative real-time PCR***

Total RNA from tissues or cultured cells was extracted using Trizol reagent according to the manufacturer’s protocol (Invitrogen, USA). cDNA was reverse-transcribed from 2 µg of total RNA using M-MLV reverse transcriptase (Takara, Japan). Semi-quantitative RT-PCR was performed using Taq polymerase. Quantitative real-time PCRs were performed with an SYBR Green Premix Ex Taq kit (Takara, Japan) on an ABI ViiA7 Real-Time PCR system (ABI, USA). All genes are expressed relative to GAPDH as an internal control and were run in triplicate. Primer sequences are listed in the Supplemental information (Supplemental Table 2).

***Western blot analysis***

The cultured cells and mouse kidney tissues were lysed in RIPA lysis buffer (Millipore) containing protease and phosphatase inhibitors (Bimake) on ice for 30 min, followed by centrifugation at 12,000 g for 10 min at 4 °C. Supernatants were separated by SDS-polyacrylamide gel electrophoresis after being quantified using a BCA assay (Takara, Japan) for normalization. Then samples were transferred to the PVDF membrane and incubated with primary antibody at 4 °C overnight after blocking with 5 % milk for 1 h. After several washes in TBS + 0.1% Tween-20, membranes were incubated with secondary antibodies (1 h at room temperature) corresponding to the respective species of primary antibodies. Proteins were detected using the ECL system (Pierce Chemical, Rockford, IL, USA), according to the manufacturer’s instructions, and revealed using a CCD camera (ImageQuantLAS4000, GE Healthcare, UK). Antibodies used in this study are summarized in Supplemental Table 3.

***Co-immunoprecipitation***

HK-2 cells or mouse kidney tissues were lysed using IP lysis buffer with 1% phenylmethanesulfonylfluoride (PMSF) supplemented with a 1% proteinase inhibitor cocktail and 1% phosphatase inhibitor on ice for 30 min. After centrifuging at 12,000 g for 10 min at 4 °C, each sample was split into two groups to incubate with 5 μg of either anti-*Glce* (anti-EGFR antibody) or mouse normal IgG as a control (Santa Cruz) overnight at 4 °C. Subsequently, protein A/G agarose (20 μL; Sigma) was added to each sample and incubated overnight at 4 °C. After centrifuging at 12,000 g for 1 min, the precipitate was washed three times with IP wash buffer and then the immunoprecipitated complex was boiled with loading buffer at 95 °C and loaded to 10% SDS-PAGE gel, followed by the immunoblotting.

***Plasmid construction and recombinant protein expression***

Virus-based overexpression and knockdown of *Glce* were conducted using the pLVX-IRES-ZsGreen1 vector and pll3.7 vector, respectively. The sh*Glce* vector was constructed by inserting the shRNA hairpin sequence into the pll3.7 vector. We designed shRNA targeting sequences *Glce* using RNAi designer online software (http://rnaidesigner.thermofisher.com/rnaiexpress/; Invitrogen) and the sequence was: GCAAGGTGTTAGGGCTCAAAT. On the other hand, the full length of the *Glce* sequence was amplified using Primer STAR HS DNA Polymerase (Takara, Japan) from cDNA and inserted into the pLVX-IRES-ZsGreen1 vector. For the IP test, a pcDNA3.1 vector with a flag tag was employed to express *Glce* in the HEK293T cells. Plasmids conveying *Glce* shRNA or *Glce* overexpression sequence and their relative vector controls were transfected into HEK293T cells with viral packaging vectors (psPAX2, pMD2G), using the calcium phosphate transfection method. Before being used to affect the HK-2 cells, the virus was harvested from the supernatant of HEK293T cells after the transfection for 48 h. The cells were not used in any experiments until they had been cultured in a complete medium without virus for at least 48 h.

***Protein-protein docking***

The X-ray structures of human *Glce* protein (h*Glce*, PDB code: 6HZZ) ^[1]^ and human inactive EGFR kinase domain (hEGFR, PDB code: 3GT8) ^[2]^ were retrieved from the RCSB Protein Data Bank for the docking calculations. For the h*Glce*, crystallographic waters, NAG, ACT, sulfate ion, calcium ion and chloride ion were removed. For hEGFR, chain B, crystallographic waters, AMP-PNP, magnesium ions were removed. Missing segments of hEGFR was built with Modeller ^[3]^ in Chimera ^[4]^, and the top-ranked model of hEGFR was chosen for next preparation. Then, the protein structures were prepared by Protein Preparation Wizard in Maestro. For each protein structure, the hydrogen atoms and the missing side chains of residues were added; the overall structure was refined with the OPLS3 force field ^[5]^. The modelled structures of h*Glce* and hEGFR were relaxed using molecular dynamic stimulation with Desmond. The simulation systems of two proteins were set up by means of Desmond System Builder in Maestro, respectively. Each protein was placed in a cubic box filled with TIP3P explicit waters and neutralized ions. The systems were then subjected to 2 ns molecular dynamic simulation with the default settings. The final relaxed structures of h*Glce* and hEGFR were submitted to ClusPro Server ^[6]^ for protein-protein docking. The best pose was selected from the conformation with the highest populated clusters and low energy.

***Adeno-Associated virus injection***

For local overexpression of *Glce* in mice kidneys, a virus packed with adeno-associated virus (AAV)-CMV-m*Glce*-GFP made by Hanbio (1 × 10^12^ vg/mL, Shanghai, China) was injected in *Glce*^-/-^ mice. For local overexpression of mutant *Glce* in mice kidneys, a virus packed with adeno-associated virus (AAV)-CMV-mut*Glce*-GFP made by Hanbio (1 × 10^12^ vg/mL, Shanghai, China) was injected in *Glce*^-/-^ mice. AAV-CMV-ZsG1-GFP (1 × 10^12^ vg/mL) was used as the control vector. All procedures applied were aseptic and mice were anesthetized with sevoflurane before surgery. The virus was orthotopically injected into the unilateral renal pelvis of *Glce*^-/-^ mice at 8 weeks of age. After the injection, the mice recovered for 6 weeks and then UUO surgery or sham operation was conducted. 2 weeks later, mice were sacrificed, and serum and tissues were collected.

***Surface plasmon resonance assays***

All experiments were performed on a Biacore T200 (GE Healthcare, Stockholm, Sweden) instrument at 25 °C using HBS-P as running buffer (10 mM HEPES, pH 7.4 containing 150 mM NaCl, 0.005% P20). *Glce* proteins were immobilized on a CM5 sensor chip (GE Healthcare) using a standard amine-coupling procedure. For interaction measurements, different concentrations of EGFR protein (extracellular part and intracellular part) and positive control polysaccharide N-S-K5 were injected into the chips. Kinetic parameters were analyzed using the 1: 1 binding model by BIACORE T200 Evaluation Software Version 1.0.

**Supplementary Figures**

**
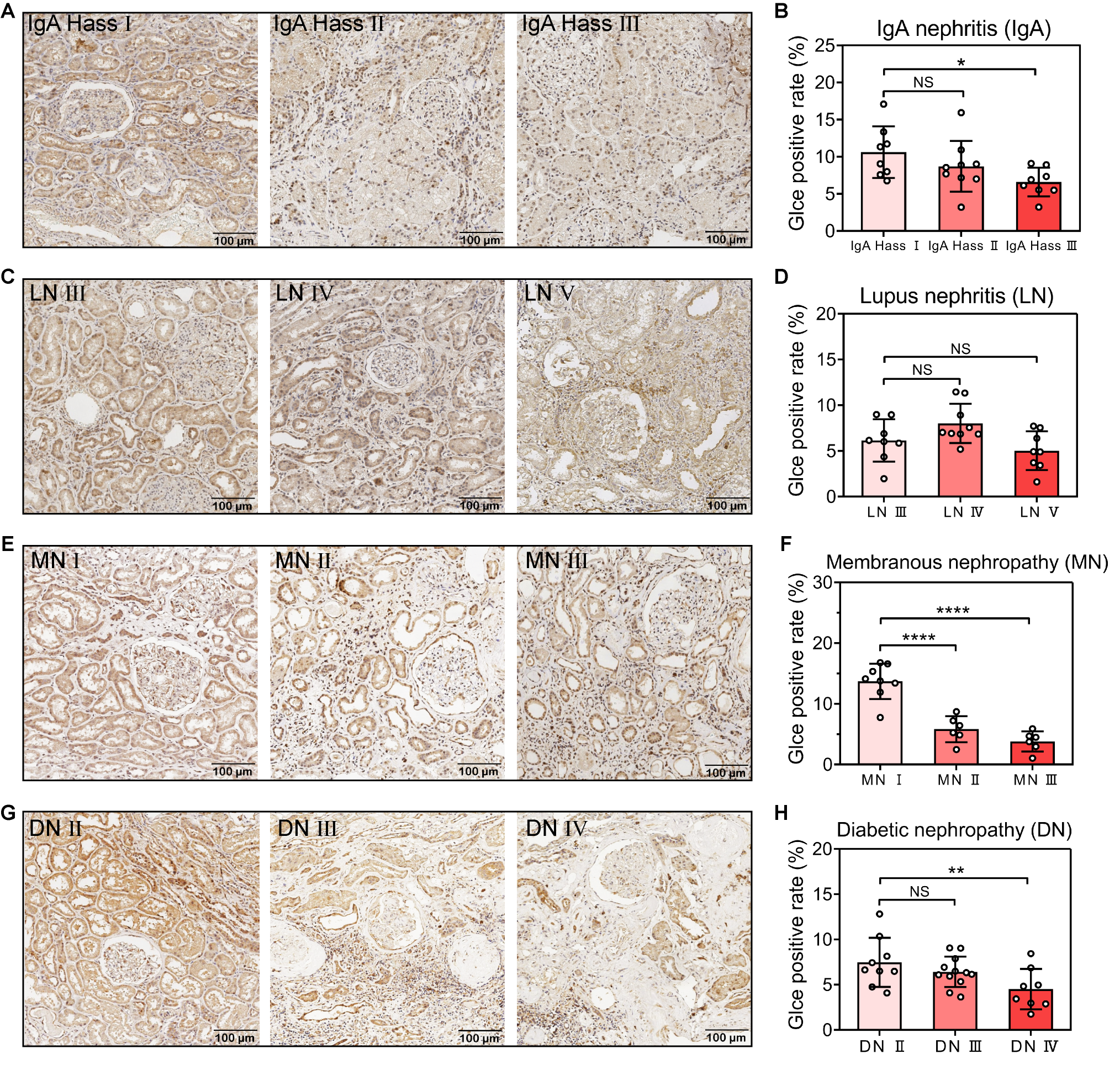
**

**Supplementary Figure S1. *Glce* expression was significantly reduced in injured human kidney.**

(**A**) Representative photomicrographs of Glce protein immunohistochemical staining (IHC) in human renal tissues from different phases of the IgA nephropathy (IgA Hass Ⅰ, n = 8; IgA Hass Ⅱ, n = 9; IgA Hass Ⅲ, n = 8). (**B**) Positive rate of Glce protein with IHC staining in the renal biopsies from patients with IgA nephropathy. (**C**) Representative photomicrographs of Glce protein immunohistochemical staining (IHC) in human renal tissues from different phases of the lupus nephritis (LN Ⅲ, n = 8; LN Ⅳ, n = 9; LN Ⅴ, n = 8). (**D**) Positive rate of Glce protein with IHC staining in the renal biopsies from patients with lupus nephritis. (**E**) Representative photomicrographs of Glce protein immunohistochemical staining (IHC) in human renal tissues from different phases of the membranous nephropathy (MN Ⅰ, n = 8; MN Ⅱ, n = 6; MN Ⅲ, n = 6). (**F**) Positive rate of Glce protein with IHC staining in the renal biopsies from patients with membranous nephropathy. (**G**) Representative photomicrographs of Glce protein immunohistochemical staining (IHC) in human renal tissues from different phases of the diabetic nephropathy (DN Ⅱ, n = 9; DN Ⅲ, n = 12; DN Ⅳ, n = 8). (**H**) Positive rate of Glce protein with IHC staining in the renal biopsies from patients with diabetic nephropathy. Scale bar: 100 μm. Data are presented as the mean ± SEM. **P* < 0.05; ***P* < 0.01; *****P* < 0.0001 by one-way ANOVA with Dunnett’s *post hoc* tests (**B**, **D**, **F** and **H**).

**
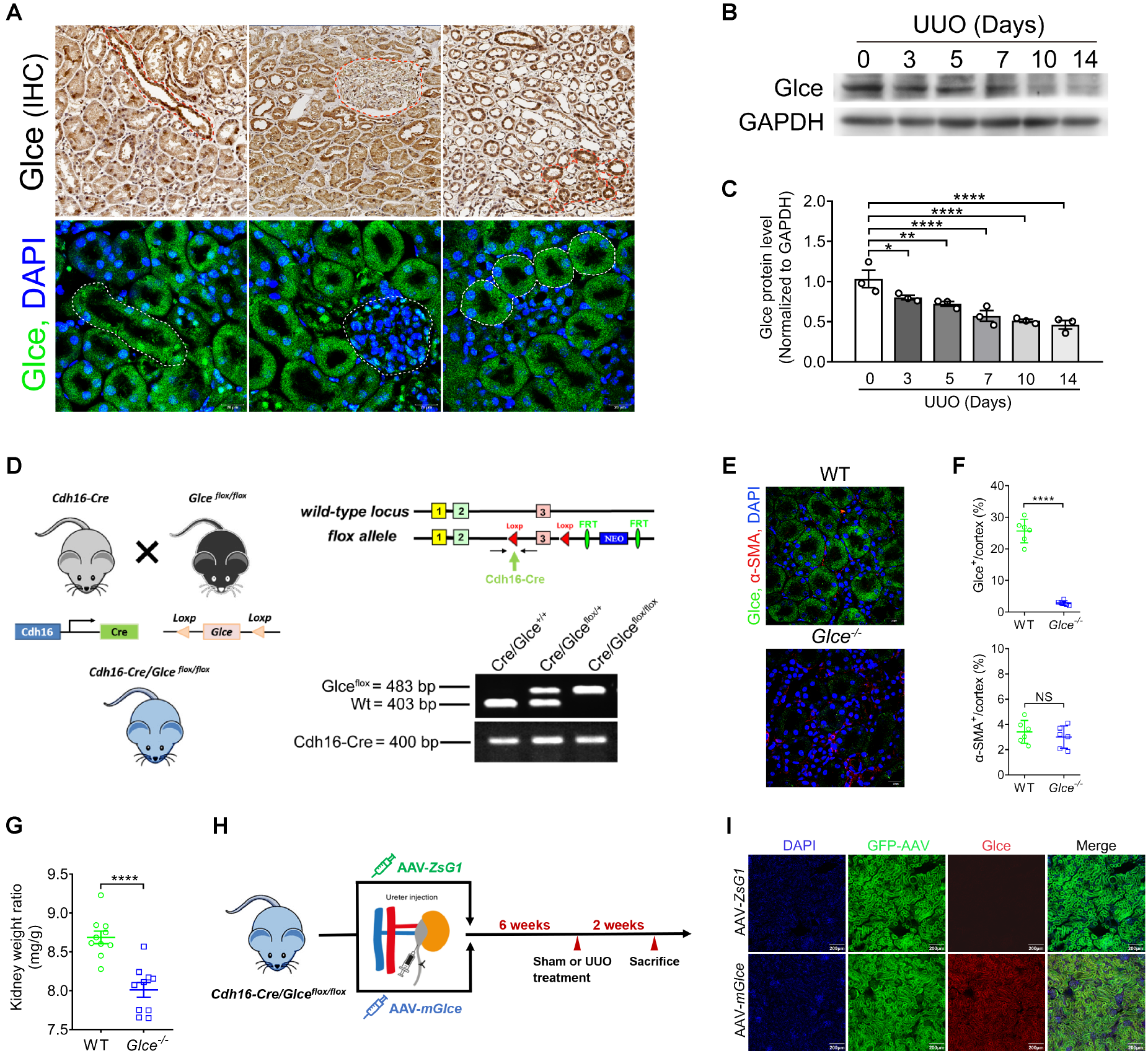
**

**Supplementary Figure S2.** ***Glce* deficiency aggravated renal failure and renal-specific AAV-mediated *Glce* overexpression improved kidney fibrosis.**

(**A**) Representative immunohistochemical staining for Glce in human renal tissues from minimal-change nephrotic syndrome (Scale bars: 100 μm) and Glce localization in WT mouse kidney by immunofluorescent staining (Scale bars: 20 μm). (**B**) Representative Western blot gel and (**C**) summarized data showing the relative protein levels of Glce in UUO-operated mice at different time points (n=3). (**D**) Experimental scheme for generating the *Cdh16-Cre/Glce^flox/flox^* mice. (**E**) Immunofluorescent staining images and (**F**) the corresponding quantification of Glce^+^(green) area/cortex (%), α-SMA^+^(red) area/cortex (%) in kidneys from WT and *Glce*^-/-^ mice(n=6). Scale bars: 20 μm. (**G**) Kidney weight/body weight ratio (%) of WT*/Glce^flox/flox^* mice (WT) and *Cdh16-Cre/Glce^flox/flox^* mice (*Glce*^-/-^) at 8 weeks of age (n=10). (**H**) Experimental scheme for AAV Orthotopic injection procedure in the renal pelvis of *Cdh16-Cre/Glce^flox/flox^* mice. (**I**) Immunofluorescence staining of Glce (red) and GFP (green) in the mice 6 weeks after AAV with full-length *Glce* injection. Scale bar: 200 μm. Data are presented as the mean ± SEM. **P* < 0.05; ***P* < 0.01; *****P* < 0.0001 by unpaired, 2-tailed Student’s t test (**F** and **G**), one-way ANOVA with Dunnett’s *post hoc* tests (**C**).


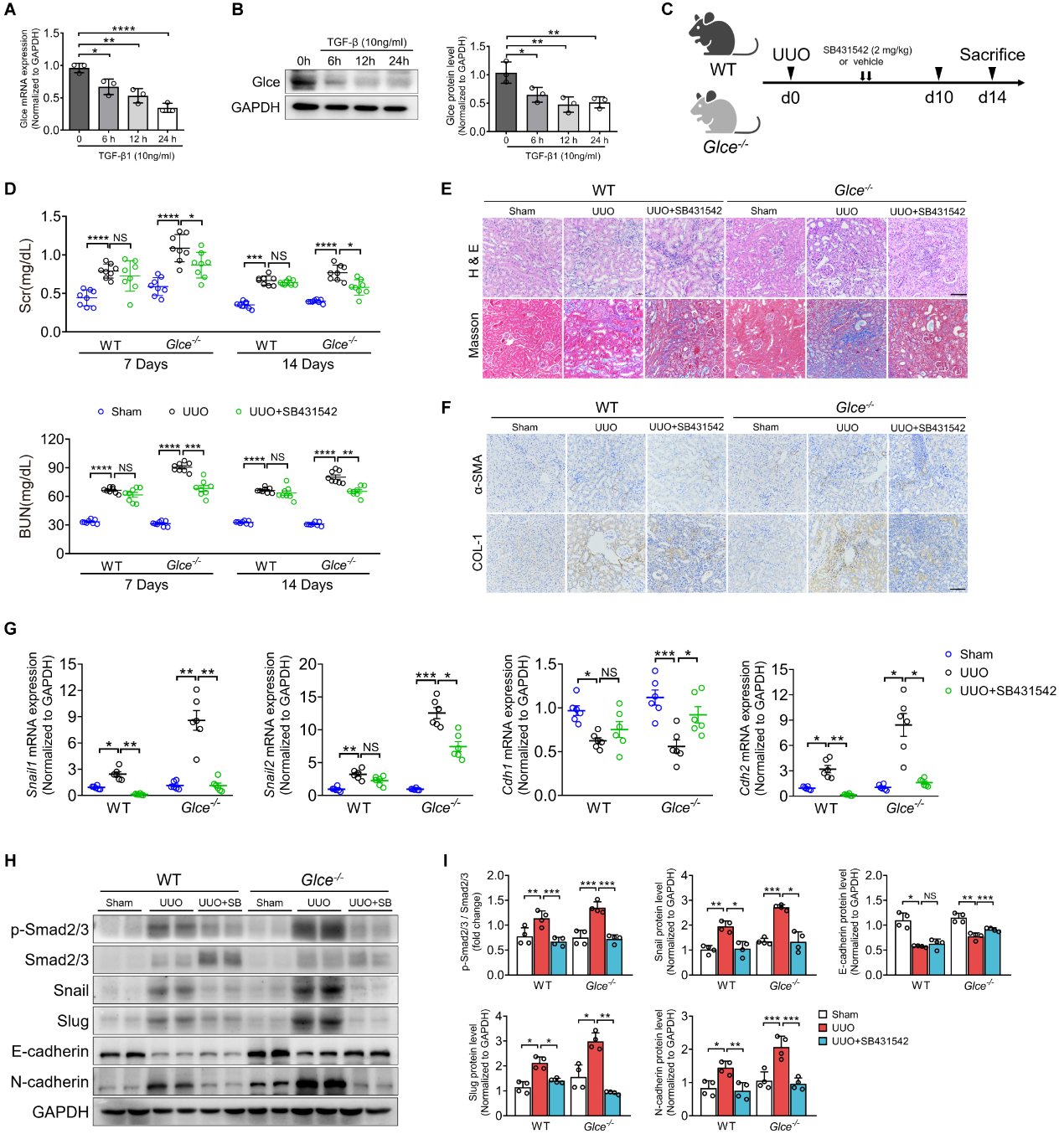


**Supplementary Figure S3. Inhibition of TGFBR1 improved UUO-induced renal fibrosis in *Glce* deficiency mice but not WT mice.**

(**A**) Relative expression of *Glce* in HK-2 cells with TGF-β1 treatment for different hours (n=3). (**B**) Protein expression levels of Glce in HK-2 cells with TGF-β1 treatment for different hours (n=3). (**C**) *In vivo* experimental setup and flowchart for the animal study. (**D**) Serum creatinine and blood urea nitrogen of WT and *Glce*^-/-^ mice following SB431542 treatment 7 days or 14 days after UUO (n=8). (**E**) Representative hematoxylin and eosin (H&E) staining, Masson staining of WT and *Glce*^-/-^ mice kidneys following SB431542 treatment 14 days after UUO. Scale bar: 100 μm. (**F**) Immunohistochemistry analysis of renal α-SMA, collagen 1 in WT and *Glce*^-/-^ mice following SB431542 treatment 14 days after UUO. Scale bar: 100 μm. (**G**) Relative mRNA expression of *Snail1, Snail2, Cdh1, Cdh2* in WT and *Glce*^-/-^ mice following SB431542 treatment 14 days after UUO (n=6). (**H**) Representative western blots of p-Smad2/3, Smad2/3, Snail, Slug, E-cadherin, N-cadherin and (**I**) protein quantification in WT and *Glce*^-/-^ mice kidneys following SB431542 treatment 14 days after UUO (n=4). Data are presented as the mean ± SEM. **P* < 0.05; ***P* < 0.01; *****P* < 0.0001 one-way ANOVA with Tukey or Dunnett’s *post hoc* tests (**A**, **B**, **D**, **G** and **I**).


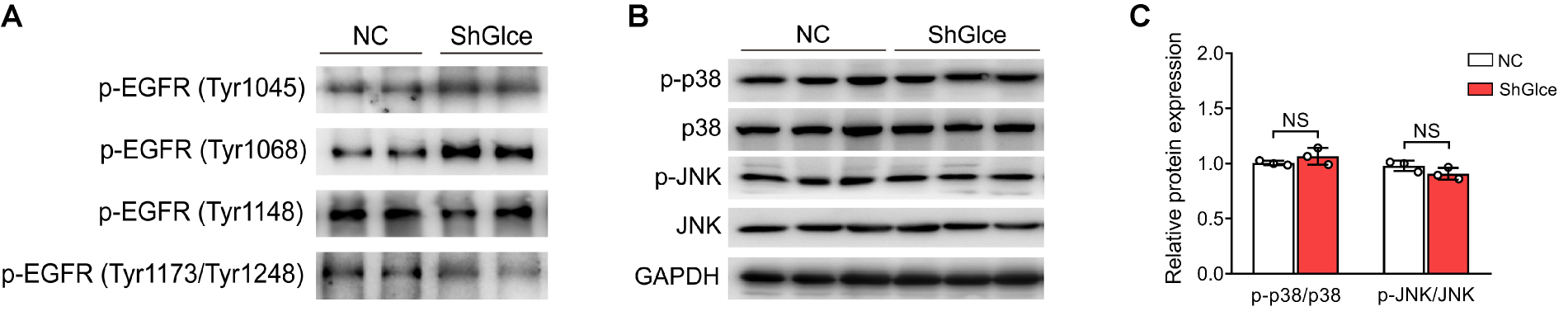


**Supplementary Figure S4. *Glce* deficiency specifically promoted EGFR phosphorylation at Tyr1068 and does not affect JNK and p-38 activation.**

1. The lysates of HK-2 cells transfected with vector or Sh*Glce* were subjected to immunoblot analysis with antibodies against phospho-EGFR (Tyr1045), phospho-EGFR (Tyr1068), phospho-EGFR (Tyr1148) and phospho-EGFR (Tyr1173/Tyr1248). (**B**) Western blots for p38, p-p38, JNK, p-JNK and GAPDH in HK-2 cells transfected with vector or ShGlce. (**C**) Quantitative densitometric analysis shows the ratio between p-p38 and total p38, and p-JNK and total JNK (n=3). Data are representative of 2 or more independent experimental replicates. Data are presented as the mean ± SEM. **P* < 0.05; ***P* < 0.01; ****P* < 0.001; *****P* < 0.0001 by unpaired, 2-tailed Student’s t test (**C**).


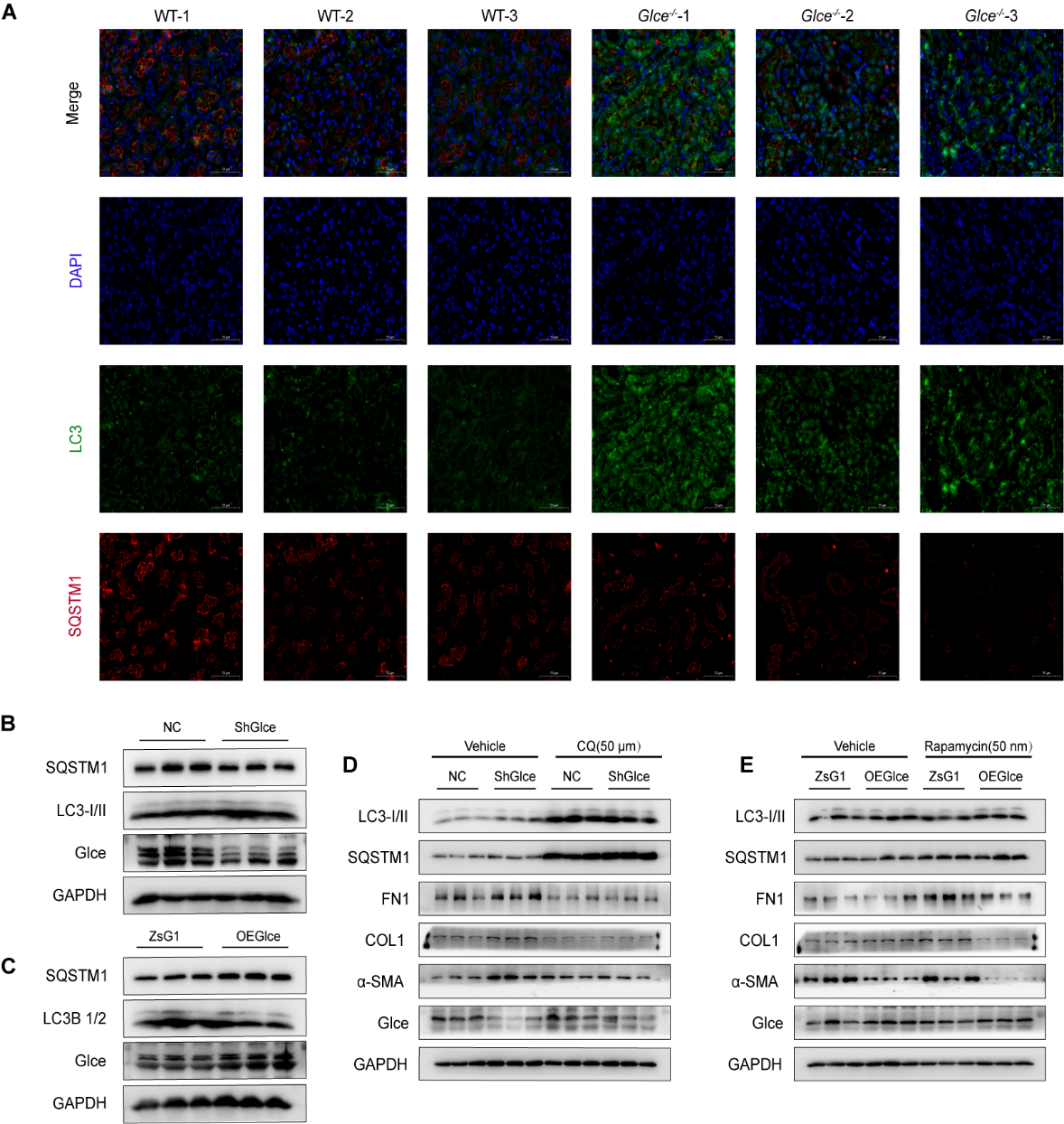


**Supplementary Figure S5. *Glce* induced autophagic changes associated with renal fibrosis.**

1. Immunofluorescence staining of microtubule-associated protein 1 light chain 3 (LC3, green) and sequestosome1 (SQSTM1, red) in kidneys of WT and Glce-/- mice. Scale bar: 50 μm. **(B, C)** Western blots showing levels LC3B I/II and SQSTM1 in HK-2 cells transfected with different plasmids (n = 3). **(D, E)** Western blot analysis of fibrosis and EMT markers in HK-2 cells transfected with different plasmids, with or without CQ or rapamycin treatment (n = 3).


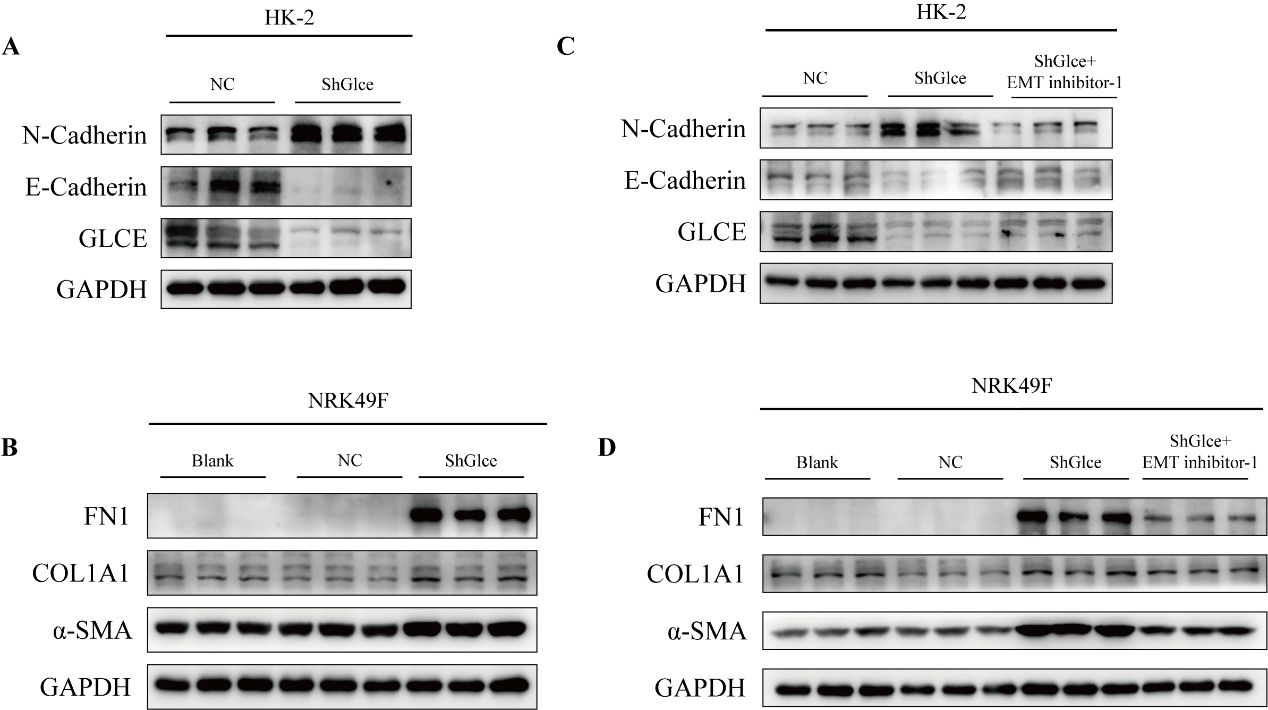


**Supplementary Figure S6. EMT enhancement driven by *Glce* deficiency in epithelial cells could partially contribute to the activation of renal fibroblasts into myofibroblasts.**

(**A, C**) Western blots showing levels Glce and EMT markers in HK-2 cells transfected with different plasmids (n = 3). (**B, D**) Western blot analysis of fibrosis markers in NRK49F cells co-cultured with HK-2 cells, with or without EMT Inhibitor-1 treatment (n = 3).

**Supplementary Table S1. Key interactions in Glce-EGFR docking model with distances**

| Key interactions | Residue in hGlce - Residue in hEGFR (Distances Å) |
| --- | --- |
| Hydrogen bonds | N255-T969(2.7Å), M402-T969(3.2Å), T349-E980(3.1Å, 3.5Å)*, T349-R975(3.0Å), N393-D984(3.3Å), R266-D984(2.8Å), W347-D982(2.9Å), S265-N747(2.9Å), R266-M983(2.8Å, 3.2Å)* |
| Salt bridges | D297-R975(2.7Å, 2.7Å)*, R266-D984(2.7Å), K264-D746(2.6Å), K299-D982 (2.5Å, 2.6Å)* |

*：The residue pair formed two hydrogen bonds or salt bridges

**Supplementary Table S2. List of Primers for Real-time PCR**

| Target | Gene ID | Primer | Sequence |
| --- | --- | --- | --- |
| GLCE  (Human) | 26035 | FP | 5'-GAAAGGAGTGGGTCTTTCAAAC-3' |
|  |  | RP | 5’- CCTTTTGCAATCAACCTAACCA -3’ |
| GAPDH  (Human) | 2597 | FP | 5'-CTGCACCACCAACTGCTTAG-3' |
|  |  | RP | 5'-AGGTCCACCACTGACACGTT-3' |
| Acta2  (Mouse) | 11475 | FP | 5'- CAGGATGCAGAAGGAGATCACAGC -3' |
|  |  | RP | 5'- AGCCACCGATCCAGACAGAGTAC -3' |
| Col1a1  (Mouse) | 12842 | FP | 5'- TGAACGTGGTGTACAAGGTC -3' |
|  |  | RP | 5'- CCATCTTTACCAGGAGAACCAT -3' |
| Col3a1  (Mouse) | 12825 | FP | 5'- GAAAGAATGGGGAGACTGGAC -3' |
|  |  | RP | 5'- TACCAGGTATGCCTTGTAATCC -3' |
| Fn1  (Mouse) | 14268 | FP | 5'- CTATAGGATTGGAGACACGTGG -3' |
|  |  | RP | 5'- CTGAAGCACTTTGTAGAGCATG -3' |
| Tgfb1  (Mouse) | 21803 | FP | 5'- CCAGATCCTGTCCAAACTAAGG -3' |
|  |  | RP | 5'- CTCTTTAGCATAGTAGTCCGCT-3' |
| Glce  (Mouse) | 93683 | FP | 5'- GGAAGTTAGGGGAAGGGTTTAA -3' |
|  |  | RP | 5'- TCTTTCGTTAGAAGATAGGCCC -3' |
| Cdh1  (Mouse) | 12550 | FP | 5'-CTCAGAAGACAGAAACGAGACT -3' |
|  |  | RP | 5'- AACCAGGTTCTTTGGAAATTCG-3' |
| Cdh2  (Mouse) | 12558 | FP | 5'- GGTGGAGGAGAAGAAGACCAGGAC-3' |
|  |  | RP | 5'-CCTCTCGTCTAGCCGTCTGATTCC -3' |
| Snai1  (Mouse) | 20613 | FP | 5'- GCTGCCTTGTGTCTGCACGAC-3' |
|  |  | RP | 5'-ACGCAGGTTGGAGCGGTCAG -3' |
| Snai2  (Mouse) | 20583 | FP | 5'-TTTTGCAGACAGATCAAACCTG -3' |
|  |  | RP | 5'- CTCCTCATGTTTATGCAGAAGC-3' |
| Gapdh  (Mouse) | 14433 | FP | 5'- AAGAAGGTGGTGAAGCAGGCATC -3' |
|  |  | RP | 5'- CGGCATCGAAGGTGGAAGAGTG -3' |

FP, Forward Primer; RP, Reverse Primer

**Supplementary Table S3. Information of antibodies**

| Antibodies | Company | Country |
| --- | --- | --- |
| Glce | Invitrogen (Cat No. PA5-97787) | USA |
| α-SMA | Sigma (Cat No. A5228) | USA |
| collagen 1 | Cell Signaling Technology (Cat No. 72026) | USA |
| Vimentin | Cell Signaling Technology (Cat No. 5741) | USA |
| Fibronectin | Cell Signaling Technology (Cat No. 26836) | USA |
| Snail | Cell Signaling Technology (Cat No. 3879) | USA |
| Slug | Cell Signaling Technology (Cat No. 9585) | USA |
| E-cadherin | Cell Signaling Technology (Cat No. 3195) | USA |
| N-cadherin | Cell Signaling Technology (Cat No. 13116) | USA |
| Smad2/3 | Cell Signaling Technology (Cat No. 8685) | USA |
| EGFR (WB/IP) | Cell Signaling Technology (Cat No. 4267) | USA |
| p38 | Cell Signaling Technology (Cat No. 8690) | USA |
| JNK | Cell Signaling Technology (Cat No. 3708) | USA |
| Phospho-JNK (Thr183/Tyr185) | Cell Signaling Technology (Cat No. 9255) | USA |
| Phospho-EGFR (Tyr1068) | Cell Signaling Technology (Cat No. 3777) | USA |
| Phospho-EGFR (Tyr1045) | Cell Signaling Technology (Cat No. 2237) | USA |
| Phospho-EGFR (Tyr1148) | Cell Signaling Technology (Cat No. 4404) | USA |
| Phospho-EGFR (Tyr1173/Tyr1248) | Cell Signaling Technology (Cat No. 2244) | USA |
| Phospho-MEK1/2 (Ser221) | Cell Signaling Technology (Cat No. 2338) | USA |
| MEK1/2 | Cell Signaling Technology (Cat No. 9126) | USA |
| Phospho-Erk1/2 (Thr202/Tyr204) | Cell Signaling Technology (Cat No. 4370) | USA |
| Erk1/2 | Cell Signaling Technology (Cat No. 4695) | USA |
| Phospho-p38 (Thr180/Tyr182) | Cell Signaling Technology (Cat No. 4511) | USA |
| Phospho-Smad2/3 | Abcam (Cat No. ab254407) | UK |
| TGF-β1 | Abcam (Cat No. ab92486) | UK |
| EGFR (IF) | Abcam (Cat No. ab30) | UK |
| Mouse IgG | Abcam (Cat No. ab6278) | UK |
| GM130 | Abcam (Cat No. ab195303) | UK |
| GAPDH | Proteintech (Cat No. 10494-1-AP) | USA |

**Supplementary References**

[1] C. Debarnot, Y. R. Monneau, V. Roig-Zamboni, V. Delauzun, C. Le Narvor, E. Richard, J. Hénault, A. Goulet, F. Fadel, R. R. Vivès, B. Priem, D. Bonnaffé, H. Lortat-Jacob, Y. Bourne, *Proc Natl Acad Sci U S A* **2019**, *116* (14), 6760, https://doi.org/10.1073/pnas.1818333116.

[2] N. Jura, N. F. Endres, K. Engel, S. Deindl, R. Das, M. H. Lamers, D. E. Wemmer, X. Zhang, J. Kuriyan, *Cell* **2009**, *137* (7), 1293, https://doi.org/10.1016/j.cell.2009.04.025.

[3] N. Eswar, D. Eramian, B. Webb, M. Y. Shen, A. Sali, *Methods Mol Biol* **2008**, *426*, 145, https://doi.org/10.1007/978-1-60327-058-8_8.

[4] E. F. Pettersen, T. D. Goddard, C. C. Huang, G. S. Couch, D. M. Greenblatt, E. C. Meng, T. E. Ferrin, *J Comput Chem* **2004**, *25* (13), 1605, https://doi.org/10.1002/jcc.20084.

[5] E. Harder, W. Damm, J. Maple, C. Wu, M. Reboul, J. Y. Xiang, L. Wang, D. Lupyan, M. K. Dahlgren, J. L. Knight, J. W. Kaus, D. S. Cerutti, G. Krilov, W. L. Jorgensen, R. Abel, R. A. Friesner, *J Chem Theory Comput* **2016**, *12* (1), 281, https://doi.org/10.1021/acs.jctc.5b00864.

[6] D. Kozakov, D. R. Hall, B. Xia, K. A. Porter, D. Padhorny, C. Yueh, D. Beglov, S. Vajda, *Nat Protoc* **2017**, *12* (2), 255, https://doi.org/10.1038/nprot.2016.169.
